# Using Bioinformatic Approaches to Identify Pathways Targeted by Human Leukemogens

**DOI:** 10.3390/ijerph9072479

**Published:** 2012-07-12

**Authors:** Reuben Thomas, Jimmy Phuong, Cliona M. McHale, Luoping Zhang

**Affiliations:** Genes and Environment Laboratory, School of Public Health, University of California, Berkeley, CA 94720, USA; Email: reuben.thomas@berkeley.edu (R.T.); j_phuong@cal.berkeley.edu (J.P.); cmchale@berkeley.edu (C.M.M.)

**Keywords:** leukemogen, pathway, Comparative Toxicogenomics Database, carcinogen, clustering

## Abstract

We have applied bioinformatic approaches to identify pathways common to chemical leukemogens and to determine whether leukemogens could be distinguished from non-leukemogenic carcinogens. From all known and probable carcinogens classified by IARC and NTP, we identified 35 carcinogens that were associated with leukemia risk in human studies and 16 non-leukemogenic carcinogens. Using data on gene/protein targets available in the Comparative Toxicogenomics Database (CTD) for 29 of the leukemogens and 11 of the non-leukemogenic carcinogens, we analyzed for enrichment of all 250 human biochemical pathways in the Kyoto Encyclopedia of Genes and Genomes (KEGG) database. The top pathways targeted by the leukemogens included metabolism of xenobiotics by cytochrome P450, glutathione metabolism, neurotrophin signaling pathway, apoptosis, MAPK signaling, Toll-like receptor signaling and various cancer pathways. The 29 leukemogens formed 18 distinct clusters comprising 1 to 3 chemicals that did not correlate with known mechanism of action or with structural similarity as determined by 2D Tanimoto coefficients in the PubChem database. Unsupervised clustering and one-class support vector machines, based on the pathway data, were unable to distinguish the 29 leukemogens from 11 non-leukemogenic known and probable IARC carcinogens. However, using two-class random forests to estimate leukemogen and non-leukemogen patterns, we estimated a 76% chance of distinguishing a random leukemogen/non-leukemogen pair from each other.

## Abbreviations

AhR: aryl hydrocarbon receptor
ALL: acute lymphocytic leukemia
AML: acute myeloid leukemia
AUC: area-under-the-curve
CAS RN: Chemical Abstracts Service Registry Numbers
COX: cyclooxygenase
FWER: family-wise error rate
HOPACH: hierarchical Ordered Partitioning And Collapsing Hybrid
HSC: hematopoietic stem cells
IARC: International Agency for Research on Cancer
LOX: lipoxygenase
MAPK: mitogen-activated protein kinase
MNCL: mononuclear cell leukemia
NTP: National Toxicology Program
NTs: Neurotrophins
Perc: tetrachloroethylene
PTGS2: prostaglandin-endoperoxide synthase 2
RoC: Report on Carcinogens
SEPEA: Structurally Enhanced Pathway Enrichment Analysis
SVM: support vector machines
TCE: trichloroethylene

## 1. Introduction

Leukemias are cancers that originate in hematopoietic stem cells (HSC) in the bone marrow [1]. They can be broadly categorized as acute or chronic, and lymphoid or myeloid, and sub-categorized by the presence of distinct, recurring cytogenetic and genetic abnormalities [2]. Additionally, leukemias are further classified by severity, immunophenotype, rate of clonal expansion, stage of differentiation, morphology, *etc.* [3]. Acute myeloid leukemia (AML) is the most common adult leukemia while acute lymphocytic leukemia (ALL) is the most common childhood leukemia; this difference is suggestive of different etiologies. In the period 2005–2009, the age-adjusted incidence rate and the age-adjusted death rates of AML were 3.6 and 2.8 per 100,000 persons per year, respectively [2]. It is estimated that in 2012, 47,150 men and women will be diagnosed with AML and 23,540 men and women will die of AML [4]. Given the incidence and poor prognosis of leukemia, particularly AML, it is important to identify leukemogens from environmental, industrial and clinical settings. 

### 1.1. Chemical Exposures Associated with Leukemia

As with other cancers, chemical exposures have been associated with leukemia. Adult AML has been associated with exposure to benzene [5,6], pesticides [7], formaldehyde [8], organic solvents [9], cigarette smoke [10,11], and possibly other agents [12]. Therapy-related AML (t-AML) accounts for 10–20% of AML cases in adults and 75% of these cases are caused by alkylating agents, with the remaining cases caused by topoisomerase II inhibitors and other agents [13]. Childhood leukemia has been associated with exposure to parental smoking, pesticides, traffic fumes, paint, and household chemicals [14]. 

Additional environmental and therapeutic agents have been identified as human leukemogens by the International Agency for Research on Cancer (IARC) [15] and the U.S. National Toxicology Program (NTP) [16], based on sufficient evidence in human studies or limited evidence in animal studies. Part of the World Health Organization, the IARC generates *IARC Monographs* identifying environmental factors (chemicals, complex mixtures, occupational exposures, physical agents, biological agents, and lifestyle factors) that can increase the risk of human cancer. Interdisciplinary working groups of expert scientists review the published studies and evaluate the weight of the evidence that an agent can increase the risk of cancer. Since 1971, more than 900 agents have been evaluated, of which more than 400 have been identified as *carcinogenic* (Group 1), *probably carcinogenic* (Group 2a), or *possibly carcinogenic* (Group 2b) to humans [15]. The NTP prepares the Report on Carcinogens (RoC), a congressionally mandated, science-based, public health report that identifies agents, substances, mixtures, or exposures (collectively called “substances”) in the environment that may increase the risk for cancer. The most recent, the 12th RoC, was released in 2011 and includes 240 listings [16]. Substances are listed in the report as either *known* or *reasonably anticipated to be human carcinogens* (equivalent to IARC Group 1 or 2).

### 1.2. Biological Pathways Involved in Leukemia

Many leukemia subtypes are characterized by recurrent structural and numerical chromosomal abnormalities. For example, t-AML following alkylating agent therapy exhibits abnormalities of chromosomes 5 and/or 7 and a complex karyotype [17,18] while t-AML following treatment with topoisomerase II inhibitors is characterized by balanced chromosomal translocations. Cooperation between mutations that activate signaling pathway genes (Class I mutations) and lead to increased cell proliferation, and mutations that inactivate hematopoietic transcription factors (Class II mutations) and interfere with hematopoietic differentiation, is thought to drive leukemogenesis [19,20]. The occurrence of at least eight different genetic pathways to therapy-related myelodysplastic syndrome (t-MDS) and t-AML, defined by the combinations of specific abnormalities present in each, were proposed [21,22]. Identical abnormalities are seen in t-AML and *de novo* AML, albeit at different frequencies. 

The emerging patterns of cooperating abnormalities [23] and mutually exclusive mutations [24] suggest that a limited number of critical pathways is targeted in leukemogenesis. Analysis of global mRNA expression, microRNA expression [25,26,27], and DNA methylation [28] signatures have revealed pathways involved in AML development [29,30,31]. Chromosomal, genetic, epigenetic, gene expression and other molecular alterations in leukemia likely converge at the level of protein function and cell signaling pathways. Indeed, the biology of AML in individual patient peripheral blood samples can be quantitatively characterized at the protein level using single-cell network profiling of specific pathways [32,33]. AML pathways include the nuclear factor kappa-B [34], mitogen-activated protein kinase (MAPK) [35], Wnt/β-catenin [36,37,38], PI3K/Akt/mTOR [39], Ras/raf/MEK/ERK [40] and aryl hydrocarbon receptor (AhR) signaling pathways [41]. Altered immune response pathways and inflammation are thought to influence leukemia progression [14,42]. 

### 1.3. Biological Pathways Targeted by Leukemogens

Limited evidence regarding the mechanisms of action of known leukemogens suggests that they target common biological pathways related to leukemogenesis. Benzene, an established human leukemogen, induces many of the specific abnormalities associated with the genetic pathways proposed for t-AML and *de novo* AML [21,22,43]. Both benzene and formaldehyde cause leukemia-specific chromosomal changes in the peripheral blood hematopoietic progenitors of otherwise healthy exposed workers [44,45]. Benzene is thought to target critical genes and pathways in hematopoietic stem cells (HSC) and bone marrow stromal cells, through the induction of genetic, chromosomal or epigenetic abnormalities, and genomic instability [46]. Pathways and biological processes such as apoptosis, proliferation, differentiation, oxidative stress, AhR dysregulation and reduced immunosurveillance, are thought to be involved in benzene-induced leukemogenesis. We recently reported altered expression of genes in immune response, inflammatory response, oxidative phosphorylation, and the AML pathway in the peripheral blood of workers occupationally exposed to a range of benzene levels [47]. Altered expression of genes related to mitochondria, oxidative phosphorylation, oxidative stress response, ribosomes, and DNA repair, was observed several months to years before development of clinically overt disease in patients who developed t-MDS/AML following chemotherapeutic regimens for lymphoma [48]. 

### 1.4. Study Aim

We hypothesized that common biological pathways involved in hematopoiesis and leukemogenesis would be enriched in toxicogenomic data from people exposed to leukemogens, and that distinct pathways would be enriched in those exposed to subtypes of leukemogens, such as alkylating agents. Analysis of altered pathways in human toxicogenomic data has been proposed as a basis to classify carcinogens [49] and pathway analysis of such data from the CTD [50] has been used to identify chemical-disease relationships [51]. Around 250 annotated human biochemical pathways are curated in the Kyoto Encyclopedia of Genes and Genomes (KEGG) database [52,53,54]. The goals of the current study were as follows: (1) to identify common KEGG pathways targeted by human leukemogens identified from IARC Monographs and NTP’s 12th RoC, through pathway analysis of genes and proteins reported in CTD; (2) to investigate whether different subtypes of leukemogens (based on their known mechanisms of action) would target distinct pathways; and, (3) to determine whether human leukemogens could be distinguished from carcinogens that were unknown to be associated with human leukemia (non-leukemogenic carcinogens) using their identified targeted pathways.

## 2. Results and Discussion

### 2.1. Identification of Leukemogens and Non-Leukemogenic Carcinogens

**Figure 1:**
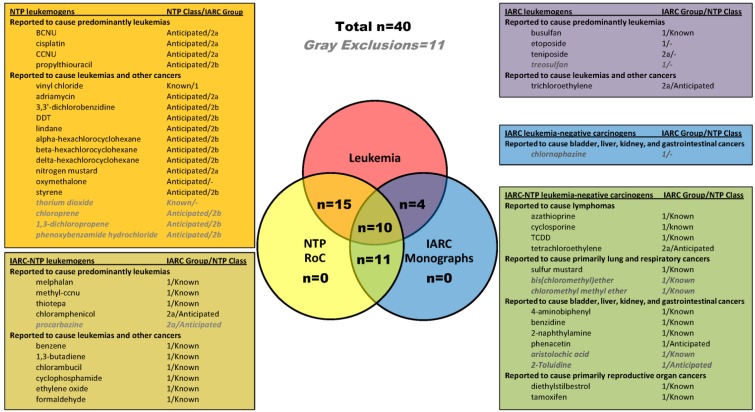
Human leukemogens and non-leukemogenic carcinogens identified from NTP and IARC reports. The Venn diagram shows the numbers of leukemogens (n = 29) and non-leukemogenic carcinogens (n = 11) identified from IARC and NTP, for which CTD data were available. The boxes detail the IARC and NTP carcinogen classifications of all 35 leukemogens and all 16 non-leukemogenic carcinogens, sorted by agency from which they were selected. Within the boxes, the chemicals are organized by reported disease associations, then by IARC group number, then alphabetically by name. “-” group or class indicates no report available. The 11 chemical names for which no CTD data were available are shown in gray-italics.

From chemicals classified as carcinogens by IARC (Group 1 and 2a) and NTP (Known and reasonably Anticipated), we identified known and probable carcinogens that were associated with leukemia in humans (n = 35), and a group of carcinogens that currently were not known to be associated with leukemia (n = 16), based on the conclusions of each agency. For the purposes of this study, we considered the first group as “leukemogens” and the second group as“non-leukemogenic carcinogens”. The carcinogen classification status of each chemical according to both IARC and NTP are listed in Figure 1 and detailed in supplementary material, Table S1 (leukemogens) and Table S2 (non-leukemogenic carcinogens). Definitions of the disease abbreviations used in Tables S1 and S2 are provided in supplementary material, Table S3. All carcinogens identified (both leukemogens and non-leukemogenic carcinogens), regardless of CTD data availability, are listed in the boxes in Figure 1, with the box colors coordinated with the Venn diagram segments. The chemicals for which CTD data were available, are listed in gray, italicized text, within each box. Of the 35 human leukemogens identified, CTD data were available for 29 leukemogens (10 from both NTP and IARC, 15 from NTP only, 4 from IARC only). Of the 16 non-leukemogenic carcinogens, CTD data were available for 11; these chemcials were selected as non-leukemogenic based on data from both IARC and NTP.

The characteristics of each chemical, such as CAS RN, environmental/therapeutic status, associated cancers, and purported mechanism of action, are listed in Table S1(a–c) (leukemogens) and Table S2(a,b) (non-leukemogenic carcinogens). Among the 29 leukemogens for which CTD data were available, 13 are environmental or industrial chemicals and 16 are therapeutic agents. Regarding mechanism of action, eight are alkylating agents (BCNU, busulfan, CCNU, chlorambucil, cyclophosphamide, melphalan, methyl-CCNU, and thiotepa); three are adduct-forming chemicals (1,3-butadiene, cisplatin, vinyl chloride, and ethylene oxide); one induces chromosome abberations (benzene); 1 has alkylating and DNA adduct-forming properties (ethylene oxide); one has DNA adduct-forming and chromosome abberation inducing properties (formaldehyde); two are topoisomerase II inhibitors (etoposide, teniposide); three have miscellaneous effects (propylthiouracil, antithyroid agent; adriamycin, antimitotic effects; oxymetholone, synthetic androgen); and 10 have unknown mechanism of action. Though several of the leukemogens were predominantly associated with leukemia, many were also associated with other cancers, as listed in Table S1(a–c).

Among the 11 non-leukemogenic carcinogens for which CTD data are available, eight are environmental or industrial chemicals and three are therapeutic agents. Two of the chemicals are adduct forming (2-naphthylamine and benzidine); TCDD acts through the AhR to modify gene expression; azathiprine induces DNA damage by 6-thioguanine accumulation in DNA; and the remaining chemicals have unknown mechanisms of action. These 11 non-leukemogenic carcinogens are predominantly associated with either lymphoma; lung and respiratory cancers; bladder, liver, kidney, and gastrointestinal cancers; or reproductive organ cancers, as shown in Figure 1 and detailed in supplementary Table S2(a,b). 

One limitation of the current study is that we selected the leukemogens and non-leukemogenic carcinogens from data reported by two carcinogen classification agencies, the IARC and the NTP, which have different goals and procedures. In addition, the strength of the association of each chemical with leukemia varies in the studies supporting IARC and NTP classifications; thus the possibility of misclassification exists. Further, we identified leukemogens based on evidence from human data only, which may be limited for some chemicals, such as those that have been banned. We focused on human data because differences in the metabolism of, and response to xenobiotic exposures, between animals and human have been reported. For example, the proportions of benzene metabolites produced differ among mice, rats and humans [55] and the types of leukemia induced differs in rodents and humans [16,56,57]. Finally, one of the major rat strains used in xenobiotic animal studies, F344 rat, has a high background rate of developing a specific type of leukemia [58].

### 2.2. Enrichment of KEGG Pathways in Genes and Proteins Associated with Leukemogens and Non-Leukemogenic Carcinogens

**Figure 2:**
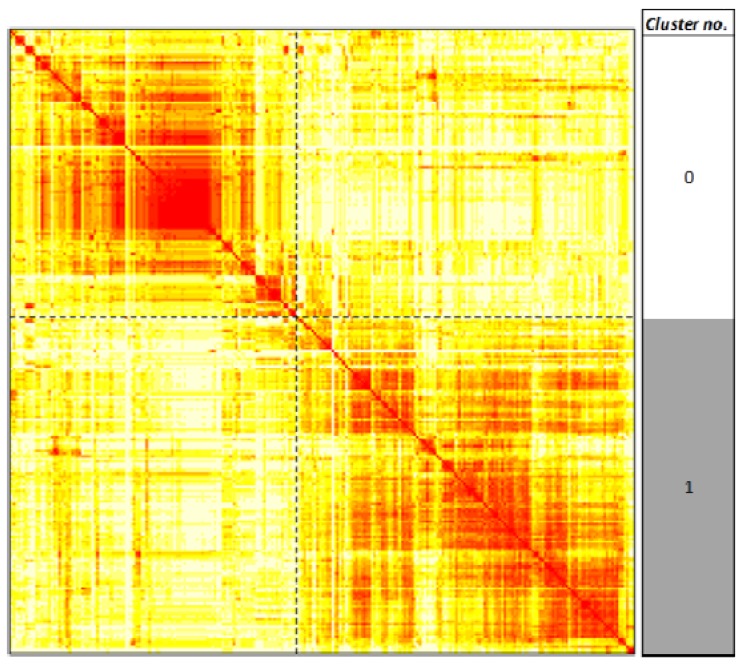
Unsupervised clustering of KEGG human pathways targeted by 29 leukemogens and 11 non-leukemogenic carcinogens. The 250 pathways are clustered based on the distance between the columns (corresponding to the pathways) of the matrix of transformed pathway enrichment *p* values over all the 40 chemicals (corresponding to the rows). The figure is a visual representation of the distance matrix between all the chosen pathways. The color of the *(i,j)th* position of the distance matrix, where *i* and *j* represent indices in the set of human pathways indexed by values in the set {1, 2,…250}, is a measure of how close pathway *i* and pathway *j* are to each other based on the enrichment of their gene targets on all the 40 chemicals. The color ranges from white to red, with red indicating greater closeness of a pair of pathways. Dashed black lines indicate boundaries of clusters of pathways as determined by the Hierarchical Ordered Partitioning And Collapsing Hybrid (HOPACH) algorithm [60]. Two clusters, labeled 0 and 1, were identified.

We hypothesized that known leukemogens would target common pathways. To test this, we analyzed for enrichment of all 250 KEGG pathways [52,53,54] using the SEPEA algorithm [59] in human toxicogenomic data on the 29 leukemogens and 11 non-leukemogenic carcinogens, extracted from the CTD. Unsupervised clustering of these results produced two clusters (Figure 2). The probabilities of each of the 250 pathways belonging to either one of these two clusters are listed in supplementary Table S4. Cluster 0 includes 115 pathways, targeted by an average of 3 (2) out of 29 (11) leukemogens (non-leukemogenic carcinogens), while Cluster 1 includes 135 pathways, targeted by an average of 11 (5) out of 29 (11) leukemogens (non-leukemogenic carcinogens). This suggests firstly that the pathways in Cluster 1 are apparently the main targets of both the leukemogens and the non-leukemogenic carcinogens. This is contrary to the hypothesis that the pathways targeted by the leukemogens and the non-leukemogenic carcinogens would separate out. In spite of this, the average percentage of non-leukemogens targeting the pathways in Cluster 0, ~20%, is marginally higher than that for the leukemogens, ~10%. Thus, a systematic learning approach aimed at accurately distinguishing the leukemogens from the non-leukemogenic carcinogens could gain (as will be seen later) information from the pathways in Cluster 0.

Pathway analysis of data from human studies involving exposure to leukemogens would be expected to reveal changes in pathways that would create a permissive environment for the development of leukemia, such as apoptosis, oxidative stress, immune response, and inflammation, rather than the pathways targeted by specific mutations that occur in rare hematopoietic stem or progenitor cells. The top 10 KEGG biochemical non-disease and disease pathways affecting the largest number (and percentage) of leukemogens affected, with a family-wise error rate (FWER) cut off 0.01, are listed in Table 1. The probabilities of membership in either cluster are also listed. These pathways lie in Cluster 1 with relatively high cluster probabilities. Many of the leukemogen-associated biochemical pathways in Table 1 have been previously implicated in leukemogen exposure and/or leukemia. The targeting of the *Metabolism of xenobiotics by cytochrome P450* pathway, by 20 of the 29 leukemogens, is not surprising since many chemicals (e.g., benzene *etc.*) are metabolized into more toxic forms by these enzymes. Involvement of the *Glutathione metabolism* pathway by 18 of the 29 chemicals suggests that oxidative stress, shown to be involved in AML and MDS [61], may be a common mechanism of leukemogens. *Apoptosis, MAPK signaling, Toll-like receptor signaling and B and T cell receptor signaling* were all identified as pathways targeted by benzene in our recent toxicogenomic study of gene expression in 125 occupationally exposed workers [47]. *TP53* is the mostly commonly mutated gene in many cancers and the P53 tumor suppressor protein is involved in multiple cellular processes, including transcription, DNA repair, genomic stability, senescence, cell cycle control and apoptosis. In a previous analysis of pathways underlying disease, the *p53* pathway along with *ErbB* and cell cycle, characterized the cancer cluster [51]. *p53* mutations and alterations have been implicated in AML [22,62].

**Table 1:** Top 10 biological and disease KEGG pathways targeted by leukemogens and their respective membership probabilities in the two clusters in Figure 1

Pathway	No. (%) of Leukemogens	Cluster 0 Probability	Cluster1 Probability
*Biological Pathway*			
Metabolism_of_xenobiotics_by_cytochrome_P450	20 (69)	0	1
Neurotrophin_signaling_pathway	19 (66)	0	1
Glutathione_metabolism	18 (62)	0.02	0.98
Apoptosis	18 (62)	0.01	0.99
MAPK_signaling_pathway	17 (59)	0	1
Toll-like_receptor_signaling_pathway	17 (59)	0	1
p53_signaling_pathway	16 (55)	0.11	0.89
Retinol_metabolism	15 (52)	0.02	0.98
Bile_secretion	15 (52)	0.05	0.95
ErbB_signaling_pathway	15 (52)	0	1
*Disease Pathway*			
Pathways_in_cancer	23 (79)	0	1
Prostate_cancer	20 (69)	0.14	0.86
Colorectal_cancer	20 (69)	0	1
Bladder_cancer	19 (66)	0.1	0.91
Melanoma	19 (66)	0	1
Pancreatic_cancer	18 (62)	0.09	0.91
Chronic_myeloid_leukemia	18 (62)	0.01	1
Amyotrophic_lateral_sclerosis_(ALS)	18 (62)	0	1
Small_cell_lung_cancer	18 (62)	0.07	0.93
Toxoplasmosis	17 (59)	0	1

A number of disease-related (mainly cancers) pathways were also targeted by ~60% of the leukemogens in the present study (Table 1), suggesting that common mechanisms may underlie the development of cancer and leukemia. Infectious diseases such as toxoplasmosis, HTLV_1 infection, tuberculosis, measles, *etc.*, were also targeted, probably through modulation of immune response and myelotoxicity (supplementary material, Table S4).

While many of the pathways make sense in the context of the current understanding of leukemia development, our findings have identified additional pathways of potential interest with less well-known associations with leukemia. Neurotrophins (NTs) and their receptors play a key role in neurogenesis and survival. Thus, a link between the n*eurotrophin signaling* pathway and leukemia is at first surprising. However, a 2009 study of cell-surface expression in leukemic blasts of 94 acute leukemia patients identified a role for NT receptors in leukemogenesis [63]. *Retinol metabolism* was among the top 10 pathways associated with the leukemogens. Retinol metabolism was previously found to be associated with hormonally regulated cancers in an analysis of disease pathways [51]. Retinol (vitamin A) and its biologically active metabolites are essential signaling molecules that control various developmental pathways and influence the proliferation and differentiation of a variety of cell types. The retinoid signaling pathway is often compromised in carcinomas and various tumors. Disruption of the physiological actions of retinoids through mutations in *RARalpha*, one of the retinoic acid receptors, via the PML-RARalpha fusion proteins, result in acute promyelocytic leukemia (APL) [64]. Interestingly, all-trans retinoic acid (ATRA) combined with anthracycline-based chemotherapy, is the current standard treatment for APL and has increased the prognosis for this disease [65]. ATRA specifically targets the PML-RARα transcripts characteristic of the majority of APL patients, releases the dominant transcription repressor, and induces specific differentiation of promyelocytes.

### 2.3. Unsupervised Clustering of Leukemogens

**Figure 3:**
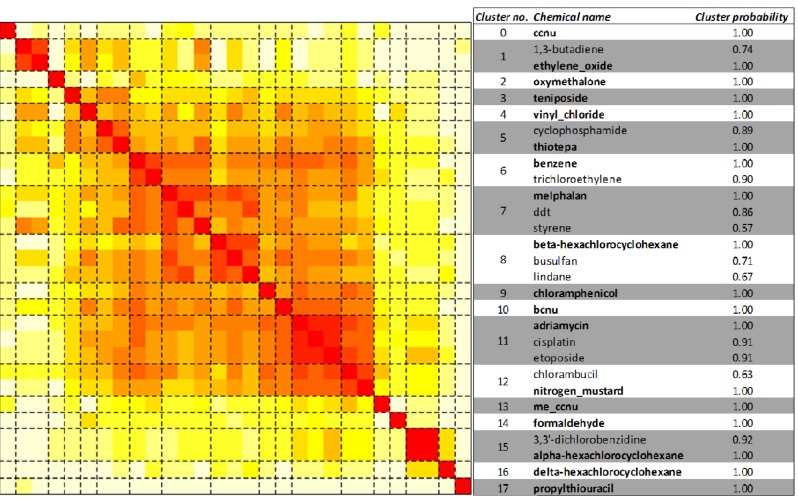
Unsupervised clustering of leukemogens. The 29 leukemogens are clustered based on the distance between rows (corresponding to the leukemogens) of the matrix of transformed pathway enrichment *p* values over all the 250 KEGG human pathways (corresponding to the columns). The figure is a visual representation of the distance matrix between all the chosen leukemogens. The color of the *(i,j)th* position of the distance matrix, where *i*,*j* represent indices in the set of 29 leukemogens indexed by values in the set {1, 2,…29}, is a measure of how close leukemogen *i* and leukemogen *j* are to each other based on the enrichment of their gene targets on all the KEGG human pathways. The color ranges from white to red, with red indicating greater closeness of leukemogen pairs. Dashed black lines indicate the boundaries of clusters of leukemogens as determined by the HOPACH algorithm [60]. Eighteen clusters, labeled from 0 to 17, were identified. Listed on the right are the chemical names, the medoid chemicals (or chemicals with pathway response pattern most similar to other chemicals in the cluster) for each cluster identified in bold case, and the cluster membership probabilities of each of the leukemogens.

We hypothesized that subtypes of leukemogens would target distinct pathways. Unsupervised clustering of the 29 leukemogens by their associated pathways produced 18 clusters, comprising 1 to 3 chemicals, as shown in Figure 3. The medoid leukemogen of each cluster, the leukemogen that best represents the pathway enrichment pattern of all others leukemogens assigned to that cluster, is also shown in Figure 3, as well as cluster membership probabilities for all 29 leukemogens. The large number of clusters and small number of leukemogens per cluster suggests a diversity of the mechanisms of action among the leukemogens. Interestingly, the three drugs used for cancer therapy—adriamycin, cisplatin and etoposide cluster together. Lindane, α-hexachlorocyclohexane, β-hexachlorocyclohexane and δ-hexachlorocyclohexane often occur as a mixture and hence the NTP or IARC reports on the carcinogenicity of the mixture. The gene interactions from CTD were, however, obtained for individual components of this mixture. Lindane and β-hexachlorocyclohexane cluster separately from α-hexachlorocyclohexane and δ-hexachlorocyclohexane. Alkylating agents or topo II inhibitors did not cluster together, as expected.

We examined whether structural similarity among chemicals explained the clustering pattern. The order of chemicals as suggested by the unsupervised clustering was not significant for structural similarity (*p* value = 0.35). Further, a heat map of the reordered distance matrix of the Tanimoto coefficients also suggests that these leukemogens are structurally diverse (supplementary material, Figure S1). The mean 2D Tanimoto coefficient between a pair of leukemogens is 0.2 and it is less than 0.4 for 90% of all pairs of leukemogens (supplementary material, Figure S2). To the best of our knowledge, structural similarity among leukemogens has not been reported. A recent study showed that *in vitro* myelotoxicity of chemical compounds could be predicted from molecular structure using *in silico* computational modeling [66]. Since the chemical leukemogens in our study cluster independently of structure and major mechanism of action (alkylating agent, topoisomerase II inhibitor, *etc.*), it is possible that other characteristics such as less well-known mechanisms of action, underlie the cluster patterns. While one of the best known and most well studied leukemogens, benzene, has multiple potential mechanisms of action [46], many of the leukemogens included in our study have a paucity of mechanistic data and unknown mechanisms of action.

### 2.4. Distinguishing Leukemogens and Non-Leukemogenic Carcinogens

We sought to determine whether pathway analysis could distinguish the leukemogens from the non-leukemogenic carcinogens. Such an approach could be applied to screen chemicals for leukemogenic potential or to predict individuals at increased risk of developing leukemia regardless of exposure status (e.g., exposome study [67]). We applied three bioinformatic methods to determine whether leukemogens could be distinguished from non-leukemogenic carcinogens: unsupervised clustering; one class support vector machines (SVM); and, two-class random forests. 2.4.1. Unsupervised Clustering. 

**Figure 4:**
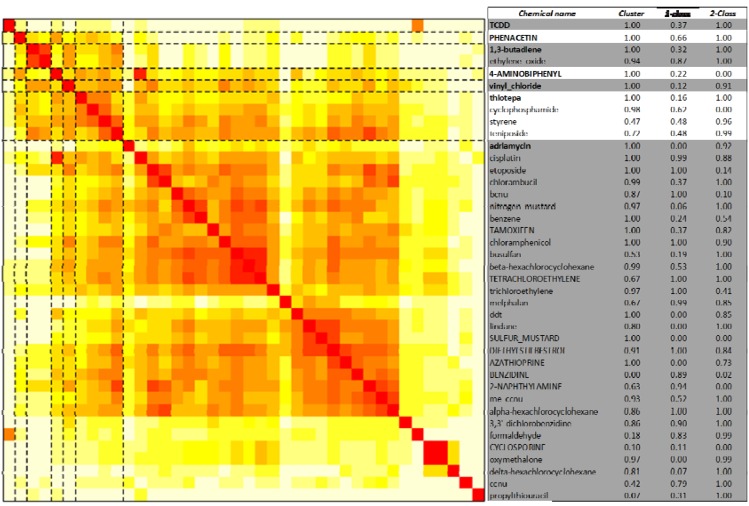
Unsupervised clustering and supervised classification of leukemogens and non-leukemogenic carcinogens. The 40 chemicals (29 leukemogens and 11 non-leukemogenic carcinogens) are clustered based on the distance between the rows (corresponding to the chemicals) of the matrix of transformed pathway enrichment *p* values over all the 250 KEGG human pathways (corresponding to the columns). The figure is a visual representation of the distance matrix between all the chosen chemicals. The color of the *(i,j)th* position of the distance matrix is a measure of how close chemical *i* and chemical *j*, where *i,j* represent indices in the set of 40 chemicals indexed by values in the set {1, 2,…40}, are to each other based on the enrichment of their gene targets on all the pathways. The color ranges from white to red, with red indicating closeness of a pair of chemicals. Dashed black lines indicate boundaries of chemical clusters as determined by the HOPACH algorithm [60]. Seven clusters, labeled from 0 to 6, were identified. The chemical names, leukemogens in lower case and non-leukemogenic carcinogens in upper case, are provided on the right of the figure. The medoid chemical (or chemical with pathway response pattern most similar to other chemicals in the cluster) for each cluster is identified in bold and the cluster membership probabilities are provided. The mean predictions from the one and two-class classification methods (Sections 2.4.2 and 2.4.3) are provided in the last two columns. A prediction value of 1 represents the leukemogen-class of chemicals and 0 represents the non-leukemogenic carcinogen class. These predictions are based on an approximately 50% false-positive rate.

Unsupervised clustering analysis of the 29 leukemogens and 11 non-leukemogenic carcinogens revealed seven clusters, with one cluster having a majority (30) of the leukemogens and non-leukemogenic carcinogens (Figure 4). The 11 non-leukemogenic carcinogens were not distinct and only five of them (benzidine, cyclosporine, tcdd, phenacetin and 4-aminobiphenyl) separated out. These clusters along with the chemical-specific cluster probabilities (supplementary material, Table S5) indicate that it is impossible (better than say using an equal probability head-tail coin toss) to separate the set of leukemogens from the set of non-leukemogenic carcinogens in an unsupervised manner. Even though the identified clusters of leukemogens in Figure 2 and Figure 3 are different; their relative order remains more or less the same. Interestingly, trichloroethylene (TCE, an IARC Group 2a carcinogen) and tetrachloroethylene (Perc, a non-leukemogenic carcinogen), to which co-exposure frequently occurs, were subclustered together. Epidemiological studies have shown TCE exposure to be associated with kidney and liver cancers as well as NHL, with weak evidence for leukemia [68,69]. In subjects exposed to Perc in drinking water, an elevated relative risk of leukemia was observed among ever-exposed subjects that increased further among subjects whose exposure level was over the 90th percentile [70] but the plausibility of this finding has been questioned [71]. It is possible that the lack of association with human leukemia is due to the limited number of exposure case studies for Perc alone. Carcinogenicity studies of Perc in several rat strains showed moderate, not clearly dose-related, increases in mononuclear cell leukemia (MCL), only in F344 rats [72]. However, latency was not decreased by exposure and the incidence in the treated groups was within the overall control range. Because the F344 rat strain is highly predisposed to developing MCL, the results were not considered predictive for human cancer risk [72]. In a series of studies conducted by the National Toxicology Program (NTP), Perc was one of five chemicals for which leukemia (used collectively to summarize multiple neoplasms including MNCL), was the only neoplastic change in both male and female rats [58].

#### 2.4.2. One-Class Support Vector Machines

The second attempt to distinguish the leukemogens from the non-leukemogenic carcinogens used a one-class support vector machines (SVM) approach to learn the pathway enrichment pattern of the leukemogen class of chemicals. The probability that a given chemical is identified as a leukemogen is estimated via a bootstrapping procedure involving five-fold cross validation, *i.e.*, the pathway enrichment pattern of 80% of the leukemogens is used to predict that of the remaining 20%. The fraction of leukemogens identified correctly and the fraction of non-leukemogens identified incorrectly across all the 1000 bootstrap steps are both around 50%. This again suggests that one-class SVM is no better than using a coin toss for our purpose. 

#### 2.4.3. Two-Class Random Forests

Our third approach to classification of leukemogens and non-leukemogenic carcinogens involved the use of random forests [73]. This analysis differs from the previous two methods in that the pathway enrichment patterns for both the leukemogen and the non-leukemogen class are learned. One class SVM involved learning only the leukemogen class patterns while the clustering process did not involve any learning. In the two-class random forest approach, the 95% confidence interval of the area-under-the-curve (AUC) of the true positive rate (fraction of correctly identified leukemogens) *versus* the false positive rate (fraction of incorrectly identified non-leukemogens) was 0.76 ± 0.07. This implies that given a random leukemogen and non-leukemogen pair, the random forest based classifier has a 76% chance of correctly distinguishing one from the other. The probability that a given chemical is identified as a leukemogen, at a false positive rate of around 50% (the same as that reported for results of one-class SVM), is estimated using information across the 1,000 bootstrap steps. These probabilities are to be interpreted in the context of the pathway enrichments of the chosen leukemogens and non-leukemogenic chemicals. Thus, the false positives characterized by relatively high probability values among the non-leukemogenic chemicals (tcdd, phenacetin, tamoxifen, diethylstilbestrol, tetrachloroethylene and azathioprine) means that their pathway enrichment patterns are more similar to that of a majority of leukemogens. This could either reflect the inadequacy of using pathways as features to distinguish between the two classes or that some of these identified false positives may actually cause leukemia. Similarly, the false negatives characterized by relatively low probability values for the leukemogens (cyclophosphamide, etoposide, bcnu, benzene and trichloroethylene) may represent atypical leukemogens. 

The top KEGG biochemical pathways driving the two-class classification, based on the largest mean decreases in gini indices, are given in Table 2. The larger this importance score of a pathway is, the better is its ability to separate the class of leukemogens from the class of non-leukemogenic carcinogens. 

**Table 2:** Top pathways that separate leukemogens from leukemogen-negative carcinogens based on mean decrease in gini index scores from the random forests based classification.

Pathway	No. (%) of Leukemogens	No. (%) of Non-Leukemogens	Cluster 0 Probability	Cluster 1 Probability	Mean Decrease Gini
Caffeine_metabolism	3 (10)	8 (73)	0.3	0.7	0.36
Arachidonic_acid_metabolism	13 (45)	6 (55)	0.03	0.97	0.2
Basal_cell_carcinoma	9 (31)	6 (55)	0.12	0.88	0.18
Drug_metabolism_other_enzymes	9 (31)	5 (45)	0	1	0.16
Ribosome	2 (7)	5 (45)	0.62	0.38	0.15
Retinol_metabolism	15 (52)	8 (73)	0.02	0.98	0.15
Metabolism_of_xenobiotics_by_cytochrome_P450	20 (69)	7 (64)	0	1	0.15
Prostate_cancer	20 (69)	7 (64)	0.14	0.86	0.14
Pentose_and_glucuronate_inter-conversions	7 (24)	5 (45)	0.89	0.11	0.14
Renin-angiotensin_system	2 (7)	3 (27)	0.37	0.63	0.13

The number (and percentage) of leukemogens and non-leukemogenic carcinogens affected (FWER cutoff of 0.01), are provided, as well as the probabilities that each of these pathways belong to one of the two clusters of pathways identified in the supplementary material, Table S4.

Compared with the pathways identified in Table 1 (leukemogens only), the pathways in Table 2 (both leukemia-positive and -negative carcinogens) in general have a relatively larger probability of being in Cluster 0 and affect a larger fraction of the non-leukemogens than the leukemogens. This suggests the differentiation of the leukemogens from the non-leukemogenic carcinogens is driven by pathways impacted by the non-leukemogenic carcinogens. *Caffeine metabolism* (mean decrease in gini index = 0.36) was the top pathway supporting the distinction between leukemogens and non-leukemogenic carcinogens, being targeted by 73% of the non-leukemogens compared with only 10% of the leukemogens. Possible inverse associations between caffeine intake and breast, liver, and colon cancer, as well as cancer of the ovary have been reported [74]. Opposing effects of caffeine and or coffee on ovarian cancer risk in postmenopausal (inverse association) [75] and premenopausal (positive association) [75,76] women, have been reported, suggesting that caffeine may be protective in a low-hormone environment. Two SNPs in the caffeine metabolizing enzyme, CYP19, (one positively and one inversely) were associated with ovarian cancer risk [77]. A common A to C polymorphism at position −163 in the CYP1A2 gene, that results in the slower metabolism of caffeine [78,79], was shown to be protective against the risk of postmenopausal breast cancer [80]. Cigarette smoking accelerates caffeine metabolism, which is mediated primarily via CYP1A2 [81]. CYP1A2 activity was also shown to be increased with increased broccoli intake and exercise [82]. A role for caffeine metabolism in hormonally regulated cancers may be what drives the distinction between leukemogens and non-leukemogenic carcinogens, but this requires further investigation.

*Arachidonic acid metabolism* was the second pathway supporting the distinction between leukemogens and non-leukemogenic carcinogens (Table 2). The first two pathways of arachidonic acid metabolism are controlled by the enzyme families cyclooxygenase (COX) and lipoxygenase (LOX). These pathways produce prostaglandins and leukotrienes, respectively, potent mediators of inflammation [83], and both pathways have been implicated in cancer [84]. Eicosanoids may represent a missing link between inflammation and cancer [85]. In our study of human occupational benzene exposure, *prostaglandin-endoperoxide synthase 2* (PTGS2 or COX2) was one of the most significant genes to be upregulated across all four doses relative to unexposed controls [47]. *PTGS2* was central to a network of inflammatory response genes impacted by benzene. The distinct roles of inflammation and the arachidonic acid metabolism pathway, as well as the ribosome, retinol metabolism, and metabolism of xenobiotics by cytochrome P450 pathways, in response to leukemogens and in leukemia and other cancers, need to be further investigated. 

#### 2.4.4. Challenges in Discriminating Leukemogens and Non-Leukemogenic Carcinogens

The analyses reported in Gohlke *et al.* [51] demonstrated that it is possible to predict chemical associations with different diseases using the pathway enrichment patterns. They also showed that diseases belonging to different classes (cancer, immune, metabolic, neuropsychiatric) can be clustered separately in an unsupervised manner. Here, we took this approach one step further by asking whether the leukemia-positive chemicals can be separated from the other known carcinogens. While two-class random forests appeared to be able to distinguish leukemia-positive and -negative carcinogens, there are some caveats to these classification approaches generally. The overlap among cancer and leukemogen pathways makes the identification of common and distinct pathways among the 250 known KEGG pathways challenging. As detailed in Table S1(a–c), many of the leukemogens are associated with one or more cancers as well as leukemia. This limits the power of the discrimination analysis making it difficult to differentiate the carcinogenic and leukemogenic effects of the leukemogens. Heterogeneity in cancer types associated with the non-leukemogenic carcinogens, in leukemia subtypes, and in the mechanisms of action of leukemogens, and associated pathways, adds an additional layer of complexity. One caveat of the two-class approach is that it assumes that the non-leukemogenic carcinogens form a class. However, the group of 11 chemicals selected in the current study is heterogeneous with respect to associated cancer types and it is unclear how well the data from the 11 non-leukemogenic carcinogens analyzed in our study could be extrapolated to other sets of non-leukemogenic carcinogens. It is also unclear how well the 29 leukemia-positive carcinogens represent the full spectrum of potential leukemia pathways. 

If our methodology were to be used for the purposes of risk assessment, the results suggest a hierarchical approach for the identification of a particular carcinogenicity hazard with the identification of leukemogens done after the chemicals were screened for other cancer types. Our study examined leukemogen pathways compared with those of non-leukemogenic carcinogens; it would be of interest to compare pathways induced by leukemogens and non-cancer disease-causing chemicals. In a study examining pathways associated with various diseases, cytochrome P450 metabolism, retinol metabolism, Jak-stat signaling, Toll-like receptor signaling, and adipocytokine signaling were identified as 5 critical pathways potentially important to disease progression from both a genetic and environmental standpoint [51]. In particular, cytochrome P450 metabolism was associated with cancers, cardiovascular disease and immune-related disorders while retinol metabolism was associated with hormonally regulated cancers. 

### 2.5. Comparison of Pathway Enrichment in CTD and in Data from a Single, Well-Designed, Toxico-Genomic Study

The CTD [86] is based on the curation of chemical-gene/protein interactions reported in the literature. Some chemicals and some genes are better studied than others. Thus, there is likely to be an inherent bias in the data used for the chemical-wise pathway enrichments, which cannot be overcome by the analyses used in the current study. In addition, even though we only analyzed human CTD data, these data were generated from various types of human cells (peripheral blood of exposed subjects, various primary and cancer cell lines), under *in vivo* or *in vitro* conditions, across different exposure durations and across different doses of the chemical. In general the conclusions are based on different significance thresholds and further conclusions from studies aimed at understanding the role of a given gene in response to a given chemical are given the same weight as those aimed at understanding the responses of a larger set of genes. Further, employment of different microarray platforms or other methodologies to measure target genes/proteins could also influence experimental results. Given these variables, we felt it was important to assess how correlated the pathway analyses based on CTD data and on data from a well-designed human toxicogenomic study, were for a given chemical. Recently, we generated transcriptomic data from the peripheral blood mononuclear cells of 125 workers exposed to a range of benzene levels in an occupational setting in which we found ~3,000 differentially expressed genes [47]. We conducted pathway enrichment analyses using statistics on whether a gene was differentially expressed in at least one of the four considered dose ranges. We compared these results to those obtained using benzene-associated gene interactions from CTD. Spearman correlation between the significance of individual pathway enrichments obtained using either data set was moderate (0.45) but significant (*p* value < 0.05). The scatter plot of the ranks of the pathways based on their enrichment *p*-values is shown in supplementary material, Figure S3. Our findings suggest that despite the limitations of CTD data, pathway analysis of CTD data is an informative approach.

## 3. Experimental Section

### 3.1. Identification of Human Leukemogens and Non-Leukemogenic Carcinogens

From chemicals classified as carcinogens by IARC (Group 1 and 2a) and NTP (Known and Reasonably Anticipated), we identified known and probable carcinogens that were associated with leukemia in humans (n = 35), and a group of carcinogens that currently were not known to be associated with leukemia (n = 16), based on the conclusions of each agency. Leukemia-positive and non-leukemogenic carcinogens were identified from all 100 volumes of IARC’s Monograph series on the evaluation of carcinogenic risks to humans, titled *A review of human carcinogens* [15] and from NTP’s 12th Report on Carcinogens (RoC) [16]. Leukemia-positive compounds were selected in three steps: (1) IARC (Group 1 and Group 2a) and NTP (“known” or “reasonably anticipated”) carcinogens; (2) single chemical carcinogens with unique Chemical Abstracts Service Registry Numbers (CAS RN); and, (3) carcinogens that are associated with leukemia risk in humans. The specific type of exposure for each leukemia-positive carcinogen was determined (e.g., environmental or therapeutic). Statistical significance (*p* value) consistently supporting the association of each chemical with cancer from multiple exposure case-studies were provided in the IARC Monographs and chemicals with of *p* < 0.05 were included in the final list (statistical data not shown). As the NTP reports do not provide statistical significance to describe the strength of association between chemicals and cancer, chemicals that were concluded by NTP to be associated with human leukemia were included without supporting statistical data. Non-leukemogen carcinogens were identified that were currently not known to be associated with leukemia, based on the conclusions of IARC and NTP. 

### 3.2. Analysis of Enrichment of KEGG Biochemical Pathways in CTD Data

The CTD [50] contains curated chemical-gene interactions. Human CTD data were available for 29 of the leukemogens (n = 35) and 11 of the non-leukemogenic carcinogens (n = 16) selected above. These data were retrieved on 10 March 2012, and were used in a pathway enrichment method called SEPEA [51,59]. All (250) human biochemical pathways in the KEGG pathway database [52,53,54] as accessed on 23 February 2012, were analyzed. SEPEA differs from other pathway enrichment methods in that it takes into account the network structure of the various pathways in the analyses—pathways where perturbed genes (as a result of treatment) are relatively close to each other in a graph/network sense are assigned more significance. The significance of a given pathway being enriched with the targets of a given chemical is reported as a *p* value. The number of chemicals affecting a given pathway is reported using a 0.01 FWER (corresponding to a 0.01/40 threshold for significance).

### 3.3. Structural Similarity between Chemicals

Structural similarity between each pair of leukemogens was determined using the 2D tanimoto coefficients (that represent the ratio of the number of shared two dimensional structural features between the pair of leukemogens to the total number of structural features defined for this pair) obtained from the PubChem database [87] on 25 April 2012. The Tanimoto Coefficient is widely applied to rank structural similarity and regarded to convey less molecular-size bias than other methods [88,89]. The CAS RN number for each of the chemicals was used for the queries. Let 
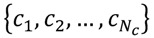
 denote a particular ordered sequence of chemicals, *O*. Let 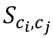 denote the 2D tanimoto coefficient between chemical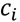 and chemical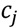. The statistic used to identify the significance of the given order of chemicals is defined as: 
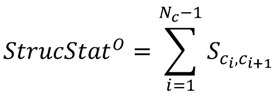
(1)

The significance of the particular order, *O*, is then estimated using a permutation test. 1,000 random permutations of the given chemicals are used to compute 1,000 
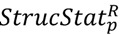
 values using Equation (1). The *p* value is then estimated by, 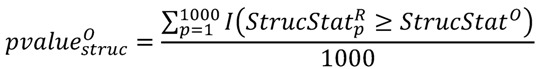
(2)

where 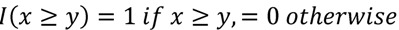


### 3.4. Unsupervised Clustering of Chemicals and Pathways


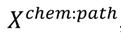
, the 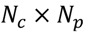 matrix of negative logarithm to the base-10 transformed SEPEA *p* values was used to identify clusters of chemicals and clusters of pathways 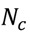. denotes the number of chemicals used (26 leukemogens or all 40 chemicals) and 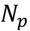 the number of human pathways (250). HOPACH [60,90] using the R [91] package *hopach* [92] was used to identify the clusters. Cosine angle was used as the distance metric. Median split silhouette was the criteria used to define the clusters and the choice of medoids (representative chemicals or pathways) for each of the clusters. The reordered distance matrix (between the 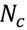
chemicals or the 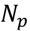 pathways) with the identified clusters was plotted as an image using the *dplot* function. The probability that a given chemical or pathway belongs to a particular cluster (as identified by the medoid chemical or pathway) was estimated via by a bootstrapping procedure as encoded in the *boothopach* function.

### 3.5. One-Class Classification of Chemicals

One class support vector machines, *svm* [93] were used to identify the pathway enrichment pattern defining the leukemogen class of chemicals from the data in the 
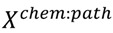
 matrix. The *svm* function in the *e1071* package [94] in R [91] was used. The radial kernel was used with the ν parameter (that is a proxy for the error rate in the training samples) set to 0.05. The 29 leukemogens were arbitrarily divided into five groups—the first group being the first six chemicals in supplementary material, Table S6, the second being the next six and so on. These groups were used in a five-fold cross-validation procedure. The pathway enrichment pattern was learnt using data from the chemicals in every four of the five groups. The *svm.predict* function was then used to predict whether the chemicals in the remaining fifth group and the 11 identified non-leukemogens are leukemogens (1) or not (0). The sampling distribution of these 0 or 1 predictions was estimated by a bootstrapping procedure similar to the one used to estimate the chemical-specific cluster probabilities in the previous section. During the *ith* of 1,000 bootstrap steps, 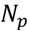
pathways were sampled with replacement from the set of all pathways. These 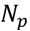 pathways were used to define the 
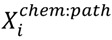
 data matrix based on the corresponding 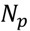 columns of 
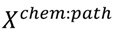
 matrix. 
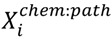
 was used to estimate the *ith* bootstrap one-class *svm* predictions of all the chemicals. 

### 3.6. Two-Class Classification of Chemicals

Random forests [73] were used to identify the pathway enrichment pattern that separates the chemicals in the leukemogen class from those in the non-leukemogenic chemical class from the data in the 
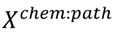
 matrix. The *CV.SuperLearner* function coded in the *SuperLearner* package [95] uses three-fold cross-validation (the percentage of leukemogens and non-leukemogenic chemicals in each fold was chosen to be more or less equal) to estimate the leukemogen class predictions for all 40 chemicals. The *CV.SuperLearner* function uses the *randomForest* package [96]. The sampling distribution of these predictions was estimated using the 1,000 random bootstrapped 
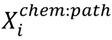
 matrices, generated as described in the previous section. The importance of each of the pathways in reducing the error of differentiating the leukemogens from the non-leukemogens was obtained from the *randomForest* function (using the entire data, 
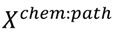
) as the corresponding mean decrease in Gini index. For each of the predictions based on the 1,000 bootstrap steps, the area-under-the-curve (AUC) of the True-Positive-Rate (fraction of leukemogens correctly identified) *versus* the False-Positive-Rate (fraction of non-leukemogens incorrectly identified) curve was estimated using the ROCR package [97].

## 4. Conclusions

We have identified common pathways targeted by single chemical human leukemogens as well as pathways that could distinguish leukemogens from non-leukemogenic carcinogens. The pathways had sufficient information to enable a reasonable separation of the leukemogens from the non-leukemogenic chemicals using a two-class classification method. As the CTD becomes populated with additional toxicogenomic datasets, our current bioinformatic approach will become more informative and discriminating, with potential applicability to the next generation of risk assessment of exposure to toxic chemicals.

## Supplementary Files

Supplementary File 1: PDF-Document (PDF, 987 KB)

## References

[1] Sawyers C.L., Denny C.T., Witte O.N. (1991). Leukemia and the disruption of normal hematopoiesis. Cell.

[2] Swerdlow S.H., Campo E., Harris N.L., Jaffe E.S., Pileri S.A., Stein H., Thiele J., Vardiman J.W. (2008). WHO Classification of Tumours of Haematopoietic and Lymphoid Tissues.

[3] Vardiman J.W. (2010). The World Health Organization (WHO) classification of tumors of the hematopoietic and lymphoid tissues: An overview with emphasis on the myeloid neoplasms. Chem. Biol. Interact..

[4] (2012). American Cancer Society. Cancer Facts & Figures 2012.

[5] Austin H., Delzell E., Cole P. (1988). Benzene and leukemia. A review of the literature and a risk assessment. Am. J. Epidemiol..

[6] Khalade A., Jaakkola M.S., Pukkala E., Jaakkola J.J.  (2010). Exposure to benzene at work and the risk of leukemia: A systematic review and meta-analysis. Environ. Health.

[7] Van Maele-Fabry G., Duhayon S., Lison D. (2007). A systematic review of myeloid leukemias and occupational pesticide exposure. Canc. Causes Contr..

[8] Goldstein B.D. (2011). Hematological and toxicological evaluation of formaldehyde as a potential cause of human leukemia. Hum. Exp. Toxicol..

[9] Albin M., Bjork J., Welinder H., Tinnerberg H., Mauritzson N., Johansson B., Billstrom R., Stromberg U., Mikoczy Z., Ahlgren T. (2000). Acute myeloid leukemia and clonal chromosome aberrations in relation to past exposure to organic solvents. Scand. J. Work Environ. Health.

[10] Sandler D.P., Shore D.L., Anderson J.R., Davey F.R., Arthur D., Mayer R.J., Silver R.T., Weiss R.B., Moore J.O., Schiffer C.A. (1993). Cigarette smoking and risk of acute leukemia: Associations with morphology and cytogenetic abnormalities in bone marrow. J. Natl. Cancer Inst..

[11] Strom S.S., Oum R., Elhor Gbito K.Y., Garcia-Manero G., Yamamura Y.  (2012). *De novo* acute myeloid leukemia risk factors: A Texas case-control study. Cancer.

[12] Cogliano V.J., Baan R., Straif K., Grosse Y., Lauby-Secretan B., El Ghissassi F., Bouvard V., Benbrahim-Tallaa L., Guha N., Freeman C. (2011). Preventable exposures associated with human cancers. J. Natl. Cancer Inst..

[13] Qian Z., Joslin J.M., Tennant T.R., Reshmi S.C., Young D.J., Stoddart A., Larson R.A., Le Beau M.M. (2010). Cytogenetic and genetic pathways in therapy-related acute myeloid leukemia. Chem. Biol. Interact..

[14] Wiemels J. (2012). Perspectives on the causes of childhood leukemia. Chem. Biol. Interact..

[15] IARC (2011). IARC Monographs on the Evaluation of Carcinogenic Risks to Humans. A Review of Human Carcinogens.

[16] NTP (2011). 12th Report on Carcinogens.

[17] Rowley J.D., Golomb H.M., Vardiman J.W. (1981). Nonrandom chromosome abnormalities in acute leukemia and dysmyelopoietic syndromes in patients with previously treated malignant disease. Blood.

[18] Smith S.M., Le Beau M.M., Huo D., Karrison T., Sobecks R.M., Anastasi J., Vardiman J.W., Rowley J.D., Larson R.A. (2003). Clinical-cytogenetic associations in 306 patients with therapy-related myelodysplasia and myeloid leukemia: The University of Chicago series. Blood.

[19] Kelly L.M., Gilliland D.G. (2002). Genetics of myeloid leukemias. Annu. Rev. Genomics Hum. Genet..

[20] Pedersen-Bjergaard J., Andersen M.T., Andersen M.K. (2007). Genetic pathways in the pathogenesis of therapy-related myelodysplasia and acute myeloid leukemia. Hematol. Am. Soc. Hematol. Educ. Program..

[21] Pedersen-Bjergaard J., Christiansen D.H., Desta F., Andersen M.K. (2006). Alternative genetic pathways and cooperating genetic abnormalities in the pathogenesis of therapy-related myelodysplasia and acute myeloid leukemia. Leukemia.

[22] Pedersen-Bjergaard J., Andersen M.K., Andersen M.T., Christiansen D.H. (2008). Genetics of therapy-related myelodysplasia and acute myeloid leukemia. Leukemia.

[23] Takahashi S. (2011). Current findings for recurring mutations in acute myeloid leukemia. J. Hematol. Oncol..

[24] Patel J.P., Gonen M., Figueroa M.E., Fernandez H., Sun Z., Racevskis J., van Vlierberghe P., Dolgalev I., Thomas S., Aminova O. (2012). Prognostic relevance of integrated genetic profiling in acute myeloid leukemia. N. Engl. J. Med..

[25] Havelange V., Stauffer N., Heaphy C.C., Volinia S., Andreeff M., Marcucci G., Croce C.M., Garzon R. (2011). Functional implications of microRNAs in acute myeloid leukemia by integrating microRNA and messenger RNA expression profiling. Cancer.

[26] Seca H., Almeida G.M., Guimaraes J.E., Vasconcelos M.H. (2010). miR signatures and the role of miRs in acute myeloid leukaemia. Eur. J. Cancer.

[27] Volinia S., Galasso M., Costinean S., Tagliavini L., Gamberoni G., Drusco A., Marchesini J., Mascellani N., Sana M.E., Abu Jarour R. (2010). Reprogramming of miRNA networks in cancer and leukemia. Genome Res..

[28] Voso M.T., D’Alo F., Greco M., Fabiani E., Criscuolo M., Migliara G., Pagano L., Fianchi L., Guidi F., Hohaus S., Leone G. (2010). Epigenetic changes in therapy-related MDS/AML. Chem. Biol. Interact..

[29] Theilgaard-Monch K., Boultwood J., Ferrari S., Giannopoulos K., Hernandez-Rivas J.M., Kohlmann A., Morgan M., Porse B., Tagliafico E., Zwaan C.M. (2011). Gene expression profiling in MDS and AML: Potential and future avenues. Leukemia.

[30] Miller B.G., Stamatoyannopoulos J.A.  (2010). Integrative meta-analysis of differential gene expression in acute myeloid leukemia. PLoS One.

[31] Mrozek K., Radmacher M.D., Bloomfield C.D., Marcucci G. (2009). Molecular signatures in acute myeloid leukemia. Curr. Opin. Hematol..

[32] Kornblau S.M., Minden M.D., Rosen D.B., Putta S., Cohen A., Covey T., Spellmeyer D.C., Fantl W.J., Gayko U., Cesano A. (2010). Dynamic single-cell network profiles in acute myelogenous leukemia are associated with patient response to standard induction therapy. Clin. Cancer Res..

[33] Cesano A., Rosen D.B., O’Meara P., Putta S., Gayko U., Spellmeyer D.C., Cripe L.D., Sun Z., Uno H., Litzow M.R. (2012). Functional pathway analysis in acute myeloid leukemia using single cell network profiling assay: Effect of specimen source (bone marrow or peripheral blood) on assay readouts. Cytometry B Clin. Cytom..

[34] Reikvam H., Olsnes A.M., Gjertsen B.T., Ersvar E., Bruserud O. (2009). Nuclear factor-kappaB signaling: A contributor in leukemogenesis and a target for pharmacological intervention in human acute myelogenous leukemia. Crit. Rev. Oncog..

[35] Towatari M., Iida H., Tanimoto M., Iwata H., Hamaguchi M., Saito H. (1997). Constitutive activation of mitogen-activated protein kinase pathway in acute leukemia cells. Leukemia.

[36] Simon M., Grandage V.L., Linch D.C., Khwaja A. (2005). Constitutive activation of the Wnt/beta-catenin signalling pathway in acute myeloid leukaemia. Oncogene.

[37] Wang Y., Krivtsov A.V., Sinha A.U., North T.E., Goessling W., Feng Z., Zon L.I., Armstrong S.A. (2010). The Wnt/beta-catenin pathway is required for the development of leukemia stem cells in AML. Science.

[38] Luis T.C., Ichii M., Brugman M.H., Kincade P., Staal F.J. (2012). Wnt signaling strength regulates normal hematopoiesis and its deregulation is involved in leukemia development. Leukemia.

[39] Altman J.K., Sassano A., Platanias L.C. (2011). Targeting mTOR for the treatment of AML. New agents and new directions. Oncotarget.

[40] Chung E., Kondo M. (2011). Role of Ras/Raf/MEK/ERK signaling in physiological hematopoiesis and leukemia development. Immunol. Res..

[41] Casado F.L., Singh K.P., Gasiewicz T.A. (2010). The aryl hydrocarbon receptor: Regulation of hematopoiesis and involvement in the progression of blood diseases. Blood Cells Mol. Dis..

[42] Shaham L., Binder V., Gefen N., Borkhardt A., Izraeli S. (2012). miR-125 in normal and malignant hematopoiesis. Leukemia.

[43] Smith M.T. (2010). Advances in understanding benzene health effects and susceptibility. Annu. Rev. Public Health.

[44] Lan Q., Zhang L., Li G., Vermeulen R., Weinberg R.S., Dosemeci M., Rappaport S.M., Shen M., Alter B.P., Wu Y. (2004). Hematotoxicity in workers exposed to low levels of benzene. Science.

[45] Zhang L., Tang X., Rothman N., Vermeulen R., Ji Z., Shen M., Qiu C., Guo W., Liu S., Reiss B. (2010). Occupational exposure to formaldehyde, hematotoxicity, and leukemia-specific chromosome changes in cultured myeloid progenitor cells. Cancer Epidemiol. Biomark. Prev..

[46] McHale C.M., Zhang L., Smith M.T. (2012). Current understanding of the mechanism of benzene-induced leukemia in humans: Implications for risk assessment. Carcinogenesis.

[47] McHale C.M., Zhang L., Lan Q., Vermeulen R., Li G., Hubbard A.E., Porter K.E., Thomas R., Portier C.J., Shen M. (2011). Global gene expression profiling of a population exposed to a range of benzene levels. Environ. Health Perspect..

[48] Li L., Li M., Sun C., Francisco L., Chakraborty S., Sabado M., McDonald T., Gyorffy J., Chang K., Wang S. (2011). Altered hematopoietic cell gene expression precedes development of therapy-related myelodysplasia/acute myeloid leukemia and identifies patients at risk. Cancer Cell.

[49] Guyton K.Z., Kyle A.D., Aubrecht J., Cogliano V.J., Eastmond D.A., Jackson M., Keshava N., Sandy M.S., Sonawane B., Zhang L. (2009). Improving prediction of chemical carcinogenicity by considering multiple mechanisms and applying toxicogenomic approaches. Mutat. Res..

[50] Mattingly C.J., Colby G.T., Forrest J.N., Boyer J.L. (2003). The Comparative Toxicogenomics Database (CTD). Environ. Health Perspect..

[51] Gohlke J.M., Thomas R., Zhang Y., Rosenstein M.C., Davis A.P., Murphy C., Becker K.G., Mattingly C.J., Portier C.J.  (2009). Genetic and environmental pathways to complex diseases. BMC Syst. Biol..

[52] Kanehisa M., Araki M., Goto S., Hattori M., Hirakawa M., Itoh M., Katayama T., Kawashima S., Okuda S., Tokimatsu T. (2008). KEGG for linking genomes to life and the environment. Nucleic Acids Res..

[53] Kanehisa M., Goto S. (2000). KEGG: Kyoto encyclopedia of genes and genomes. Nucleic Acids Res..

[54] Kanehisa M., Goto S., Hattori M., Aoki-Kinoshita K., Itoh M., Kawashima S., Katayama T., Araki M., Hirakawa M. (2006). From genomics to chemical genomics: New developments in KEGG. Nucleic Acids Res..

[55] ATSDR (2007). Toxicological Profile For Benzene. U.S. Department of Health And Human Services. http://www.atsdr.cdc.gov/toxprofiles/tp3.html.

[56] Cronkite E.P., Drew R.T., Inoue T., Hirabayashi Y., Bullis J.E. (1989). Hematotoxicity and carcinogenicity of inhaled benzene. Environ. Health Perspect..

[57] Snyder C.A., Goldstein B.D., Sellakumar A.R., Bromberg I., Laskin S., Albert R.E. (1980). The inhalation toxicology of benzene: Incidence of hematopoietic neoplasms and hematotoxicity in ARK/J and C57BL/6J mice. Toxicol. Appl. Pharmacol..

[58] Thomas J., Haseman J.K., Goodman J.I., Ward J.M., Loughran T.P., Spencer P.J. (2007). A review of large granular lymphocytic leukemia in Fischer 344 rats as an initial step toward evaluating the implication of the endpoint to human cancer risk assessment. Toxicol. Sci..

[59] Thomas R., Gohlke J., Stopper G., Parham F., Portier C.  (2009). Choosing the right path: Enhancement of biologically relevant sets of genes or proteins using pathway structure. Genome Biol..

[60] van der Laan M.J., Pollard K.S. (2003). A new algorithm for hybrid hierarchical clustering with visualization and the bootstrap. J. Stat. Plan. Inference.

[61] Hole P.S., Darley R.L., Tonks A.  (2011). reactive oxygen species play a role in myeloid leukemias?. Blood.

[62] Rucker F.G., Schlenk R.F., Bullinger L., Kayser S., Teleanu V., Kett H., Habdank M., Kugler C.M., Holzmann K., Gaidzik V.I. (2012). TP53 alterations in acute myeloid leukemia with complex karyotype correlate with specific copy number alterations, monosomal karyotype, and dismal outcome. Blood.

[63] Li Z., Beutel G., Rhein M., Meyer J., Koenecke C., Neumann T., Yang M., Krauter J., von Neuhoff N., Heuser M., Diedrich H. (2009). High-affinity neurotrophin receptors and ligands promote leukemogenesis. Blood.

[64] Mongan N.P., Gudas L.J. (2007). Diverse actions of retinoid receptors in cancer prevention and treatment. Differentiation.

[65] Mi J. (2011). Current treatment strategy of acute promyelocytic leukemia. Front. Med..

[66] Crivori P., Pennella G., Magistrelli M., Grossi P., Giusti A.M. (2011). Predicting myelosuppression of drugs from in silico models. J. Chem. Inf. Model..

[67] Smith M.T., Zhang L., McHale C.M., Skibola C.F., Rappaport S.M. (2011). Benzene, the exposome and future investigations of leukemia etiology. Chem. Biol. Interact..

[68] Wartenberg D., Reyner D., Scott C.S.  (2000). Trichloroethylene and cancer: Epidemiologic evidence. Environ. Health Perspect..

[69] Scott C.S., Chiu W.A. (2006). Trichloroethylene cancer epidemiology: A consideration of select issues. Environ. Health Perspect..

[70] Aschengrau A., Ozonoff D., Paulu C., Coogan P., Vezina R., Heeren T., Zhang Y. (1993). Cancer risk and tetrachloroethylene-contaminated drinking water in Massachusetts. Arch. Environ. Health.

[71] Ramlow J.M. (1995). Apparent increased risk of leukemia in their highest category of exposure to tetrachloroethylene (PCE) in drinking water. Arch. Environ. Health.

[72] Ishmael J., Dugard P.H. (2006). A review of perchloroethylene and rat mononuclear cell leukemia. Regul. Toxicol. Pharmacol..

[73] Breiman L. (2001). Random forests. Mach. Learn..

[74] La Vecchia C., Tavani A. (2007). Coffee and cancer risk: An update. Eur. J. Cancer Prev..

[75] Tworoger S.S., Gertig D.M., Gates M.A., Hecht J.L., Hankinson S.E. (2008). Caffeine, alcohol, smoking, and the risk of incident epithelial ovarian cancer. Cancer.

[76] Kuper H., Titus-Ernstoff L., Harlow B.L., Cramer D.W. (2000). Population based study of coffee, alcohol and tobacco use and risk of ovarian cancer. Int. J. Cancer.

[77] Kotsopoulos J., Vitonis A.F., Terry K.L., De Vivo I., Cramer D.W., Hankinson S.E., Tworoger S.S. (2009). Coffee intake, variants in genes involved in caffeine metabolism, and the risk of epithelial ovarian cancer. Canc. Causes Contr..

[78] Sachse C., Brockmoller J., Bauer S., Roots I. (1999). Functional significance of a C–A polymorphism in intron 1 of the cytochrome P450 CYP1A2 gene tested with caffeine. Br. J. Clin. Pharmacol..

[79] Han X.M., Ou-Yang D.S., Lu P.X., Jiang C.H., Shu Y., Chen X.P., Tan Z.R., Zhou H.H. (2001). Plasma caffeine metabolite ratio (17X/137X)* in vivo* associated with G-2964A and C734A polymorphisms of human CYP1A2. Pharmacogenetics.

[80] Le Marchand L., Donlon T., Kolonel L.N., Henderson B.E., Wilkens L.R. (2005). Estrogen metabolism-related genes and breast cancer risk: The multiethnic cohort study. Cancer Epidemiol. Biomark. Prev..

[81] Zevin S., Benowitz N.L. (1999). Drug interactions with tobacco smoking. An update. Clin. Pharmacokinet..

[82] Vistisen K., Loft S., Poulsen H.E. (1991). Cytochrome P450 IA2 activity in man measured by caffeine metabolism: Effect of smoking, broccoli and exercise. Adv. Exp. Med. Biol..

[83] Zeldin D.C. (2001). Epoxygenase pathways of arachidonic acid metabolism. J. Biol. Chem..

[84] Wang D., Dubois R.N. (2010). Eicosanoids and cancer. Nat. Rev. Cancer.

[85] Greene E.R., Huang S., Serhan C.N., Panigrahy D. (2011). Regulation of inflammation in cancer by eicosanoids. Prostaglandins Other Lipid Mediat..

[86] Davis A.P., King B.L., Mockus S., Murphy C.G., Saraceni-Richards C., Rosenstein M., Wiegers T., Mattingly C.J. (2011). The comparative toxicogenomics database: Update 2011. Nucleic Acids Res..

[87] Bolton E.E., Wang Y., Thiessen P.A., Bryant S.H. (2008). PubChem: Integrated platform of small molecules and biological activities. Annu. Rep. Comput. Chem..

[88] Chen X., Reynolds C.H. (2002). Performance of similarity measures in 2D fragment-based similarity searching: Comparison of structural descriptors and similarity coefficients. J. Chem. Inf. Comput. Sci..

[89] Holliday J.D., Salim N., Whittle M., Willett P. (2003). Analysis and display of the size dependence of chemical similarity coefficients. J. Chem. Inf. Comput. Sci..

[90] Van der Laan M., Pollard K. (2003). A new algorithm for hybrid hierarchical clustering with visualization and the bootstrap. J. Stat. Plan. Inference.

[91] (2009). R Development Core Team. R: A Language and Environment for Statistical Computing.

[92] Pollard K.S., Wall G., van der Laan M.J. (2010). Hopach: Hierarchical Ordered Partitioning and Collapsing Hybrid (HOPACH); R Package Version 2.10.0. http://CRAN.R-project.org/package=hopach.

[93] Schölkopf B., Platt J.C., Shawe-Taylor J., Smola A.J., Williamson R.C. (2001). Estimating the support of a high-dimensional distribution. Neural Comput..

[94] Dimitriadou E., Hornik K., Leisch F., Meyer D., Weingessel A. (2010). e1071: Misc Functions of the Department of Statistics (e1071),TU Wien; R Package Version 1.5-24. http://CRAN.R-project.org/package=e1071.

[95] Polley E.C. (2010). SuperLearner: Super Learner Prediction; R Package Version 1.1-18. http://www.stat.berkeley.edu/~ecpolley/SL/.

[96] Liaw A., Wiener M. (2002). Classification and Regression by randomForest. R. News.

[97] Sing T., Sander O., Beerenwinkel N., Lengauer T. (2009). Visualizing the Performance of Scoring Classifiers; R Package Version 1.0-4. http://CRAN.R-project.org/package=ROCR.

