# Supplementary material for: Using Bioinformatic Approaches to Identify Pathways Targeted by Human Leukemogens
Source: Int J Environ Res Public Health. 2012 Jul 12;9(7):2479–503. doi: 10.3390/ijerph9072479 (PMC3407916; doi:10.3390/ijerph9072479)
Supplement: Supplementary File 1: — PDF-Document (PDF, 987 KB) [file ijerph-09-02479-s001.pdf]

OPEN ACCESS

International Journal of
Environmental Research and Public Health
ISSN 1660-4601
www.mdpi.com/journal/ijerph

Supplementary Information

Using Bioinformatic Approaches to Identify Pathways Targeted by Human Leukemogens. *Int. J. Environ. Res. Public Health* 2012, *9*, 2479-2503

Reuben Thomas, Jimmy Phuong, Cliona M. McHale and Luoping Zhang *

Genes and Environment Laboratory, School of Public Health, University of California, Berkeley, CA 94720, USA; E-Mails: reuben.thomas@berkeley.edu (R.T.); j_phuong@cal.berkeley.edu (J.P.); cmchale@berkeley.edu (C.M.M.)

* Author to whom correspondence should be addressed; E-Mail: luoping@berkeley.edu; Tel.: +1-510-643-5189; Fax: +1-510-642-0427.

Received: 18 May 2012; in revised form: 25 June 2012 / Accepted: 26 June 2012 / Published: 12 July 2012

Figure S1. 2D Tanimoto coefficient distance matrix of the leukemogens. The figure is a visual representation of the distance matrix between the 29 leukemogens, based on structural similarity measured by 2D tanimoto coefficient, as obtained from the PubChem database (http://pubchem.ncbi.nlm.nih.gov//score_matrix/score_matrix.cgi). The order of the leukemogens in the distance matrix is the same as that determined from the unsupervised clustering analysis in Figure 3. The color of the *(i,j)*th position of the distance matrix reflects the structural similarity between leukemogen *i* and leukemogen *j*. The color ranges from white to red, with red indicating greater structural similarity. Dashed black lines correspond to boundaries of clusters of leukemogens as determined from the unsupervised clustering analysis in Figure 3.

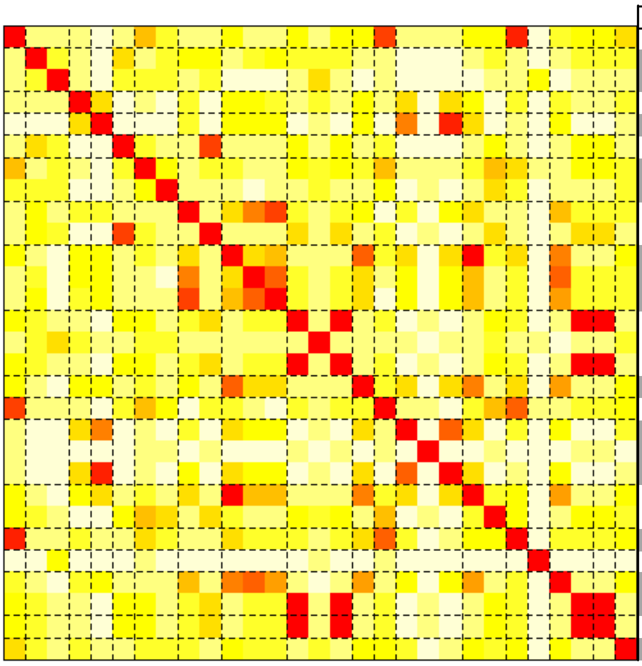

Cluster no.	*Chemical name*
0	**ccnu**
1	1,3-butadiene
	**ethylene_oxide**
2	**oxymethalone**
3	**teniposide**
4	**vinyl_chloride**
5	cyclophosphamide
	**thiotepa**
6	**benzene**
	trichloroethylene
7	**melphalan**
	ddt
	styrene
8	**beta-hexachlorocyclohexane**
	busulfan
	lindane
9	**chloramphenicol**
10	**bcnu**
11	**adriamycin**
	cisplatin
	etoposide
12	chlorambucil
	**nitrogen_mustard**
13	**me_ccnu**
14	**formaldehyde**
15	3,3'-dichlorobenzidine
	**alpha-hexachlorocyclohexane**
16	**delta-hexachlorocyclohexane**
17	**propylthiouracil**

Figure S2. Empirical distribution of 2D tanimoto coefficients. Empirical probability distribution of the 2D tanimoto coefficients obtained from each pair-wise analysis of all 29 leukemogens.

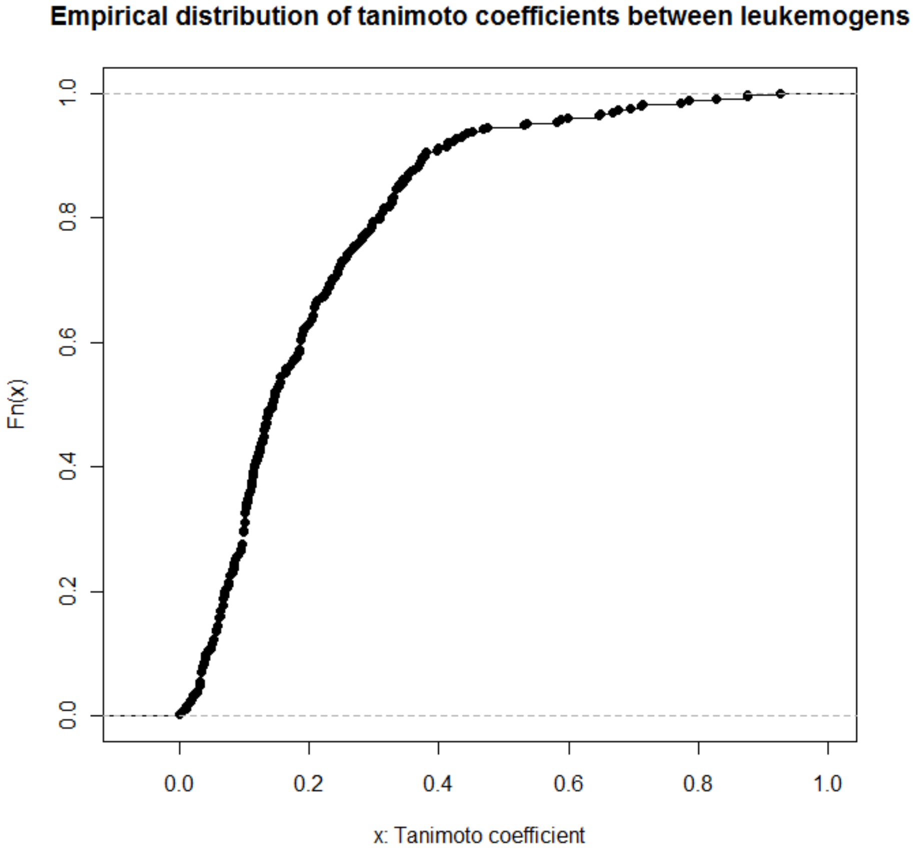

Figure S3. Correlation between benzene pathway enrichment results using gene interaction data from the Comparative Toxicogenomics Database and global gene expression profiles from a human study. Pathway enrichment analysis was computed for all 250 KEGG human pathways using the SEPEA algorithm [59] on human toxicogenomic data related to benzene from CTD and from a global gene expression study of 125 factory workers occupationally exposed to a range of benzene levels [47]. Pathways are ranked from the most significant (smallest p-values) to the least significant. The figure is a scatter plot of the ranks of the pathways based on the two data sources. The spearman correlation of the pathway enrichment values using the two data sources is 0.45 with a statistical significance of 10^{-13}.

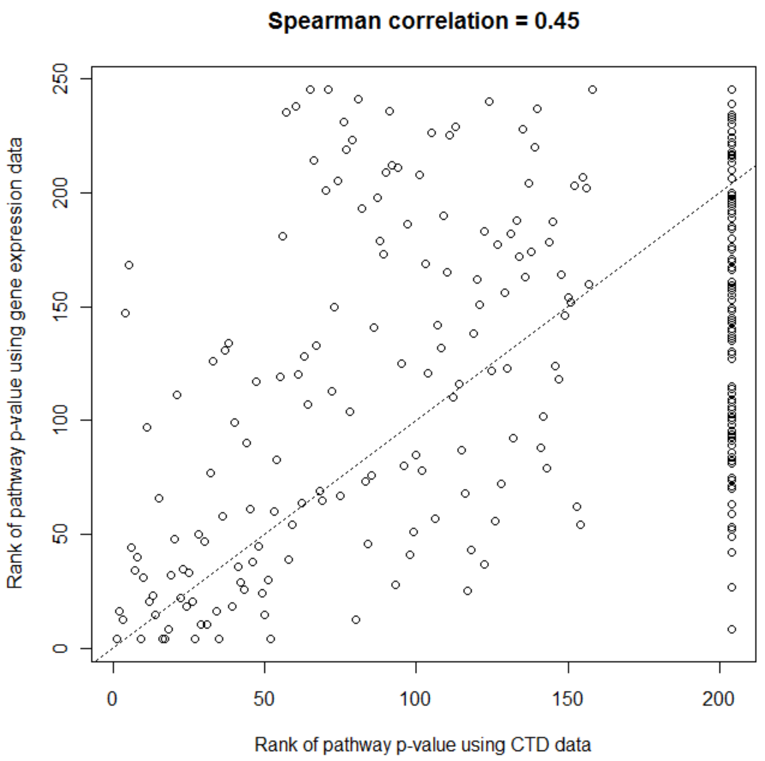

Table S1a. Chemicals found to cause leukemias in humans, according to both the IARC Monographs and the NTP 12th Report on Carcinogens.

CTD chemical name (Compound CID)	CASRN	IARC monograph chemical summaries: Chemical name	Group	Volume	Exposure type	Mode of action	Treatment use	Resultant diseases
Reported to cause predominantly leu emias								
melphalan (461602)	148-82-3	melphalan	1	9, Sup 7, 100A	M	a bifunctional alkylating agent that forms DNA crosslinks; causes clonal loss of chromosome 5 or 7	MM, STS, childhood NB, advanced ovarian adenocarcinoma, breast cancer, melanoma, polycythemia vera	**AML**, MDS
methyl-ccnu (5198)	13909-09-6	1-(2-chloroethyl)-3-(4-methylcyclohexyl) 1-nitrosourea (methyl-CCNU)	1	Sup 7, 100A	M	a bifunctional alkylating agent that induces macromolecule carbamoylation; methylates p15 promoter	HD, melanoma, hematopoietic malignancies, and cancer of the lung, gastrointestinal tract, and brain	**AML**
thiotepa (5453)	52-24-4	thiotepa	1	50, 100A	M	a trifunctional, alkylating agent	malignant lymphomas, breast cancer, ovarian cancer, meningeal carcinomatosis, bladder malignancies	**non-lymphocytic leukemia**
chloramphenicol (5959)	56-75-7	chloramphenicol	2a	50	M	-	typhoid fever, salmonellosis	**ALL**, **AML,** AA, and bone-marrow depression
procarbazine	*671-16-9 366-70-1*	*procarbazine and procarbazine hydrochloride*	*2a*	*26, Sup 7*	*M*	*generates an alkylating agent*	*HD*	*AML*
Reported to cause leukemias and oth r cancers								
benzene (241)	71-43-2	benzene	1	29, Sup 7, 100F	E/I	induces chromosome aberrations in lymphocytes.	-	**AML**, **ALL**, **CLL**, MM, NHL
1,3-butadiene (7845)	106-99-0	1,3-butadiene	1	97, 100F	E/I	-	-	**CLL**, **CML**, HD, NHL
chlorambucil (2708)	305-03-3	chlorambucil	1	26, Sup 7, 100A	M	a bifunctional alkylating agent; hallmarks of leukemia are seen as deletion or clonal loss of chromosome 5 and 7	**CLL**, GN, HD, NHL, WM, breast cancer, ovarian cancer, polycythemia vera, juvenile arthritis	**AML**, MDS, SCC, pancytopenia, melanoma
cyclophosphamide (2907)	50-18-0	cyclophosphamide	1	26, Sup 7, 100A	M	the phosphoramide mustard metabolites are active alkylating agents	**CLL**, NHL, HD, OS, MM, STS, CTCL, NB, ovarian cancer, breast cancer, burkitt's lymphoma, lymphoblastic	**AML**, MDS, bladder cancer
ethylene oxide (6354)	75-21-8	ethylene oxide	1	97, 100F	E/I	forms hydroxyethyl hemoglobin adducts with N-terminal valine, histidine, and S-cysteine	-	**AML**, **CML**, **lymphocytic leukemia,** WM, and cancer of the esophagus, stomach, brain, and breast
formaldehyde (712)	50-00-0	formaldehyde	1	88, 100F	E/I	forms DNA adducts, sister chromid exchange, micronucleii, chromosomal aberrations, and DNA cross-links in lymphocytes	-	**leukemia**, NPC, HD, MM, and cancer of the buccal cavity, larynx, pharynx, lung, kidney, skin, bladder, prostate, brain, and colon

CTD chemical name (Compound CID)	NTP RoC chemical summaries: Chemical name	Class	Exposure type	Mode of action	Treatment use	Resultant diseases
Reported to cause predominantly leu emias						
melphalan (461602)	melphalan	Known	M	alkylating agent	**CML**, MM, OS, melanoma, polycythemia vera, scleromyxedema, amyloidosis, and cancer of the ovaries, breast, testes, and prostate	**leukemia**
methyl-ccnu (5198)	1-(2-chloroethyl)-3-(4-methylcyclohexyl) 1-nitrosourea (methyl-CCNU)	Known	M	a bifunctional alkylating agent	melanoma, and cancer of the gastrointestinal tract, brain and lung	**AML**
thiotepa (5453)	thiotepa	Known	M	-	Wilm's tumor, bronchiogenic carcinoma, lymphoma, and adenocarcinoma of the breast, ovary, and bladder	**secondary AML**
chloramphenicol (5959)	chloramphenicol	Anticipated	M	-	typhoid fever, meningitis, superficial ocular infections	**secondary leukemia**
procarbazine	*procarbazine and procarbazine hydrochloride*	*Anticipated*	*M*	*-*	*HD*	*leukemia, and cancer of the soft tissue, cartilage, and bone*
Reported to cause leukemias and oth r cancers						
benzene (241)	benzene	Known	E/I	-	-	**AML**, **CLL**
1,3-butadiene (7845)	1,3-butadiene	Known	E/I	epoxide metabolites adduct DNA and form *hprt* mutations in lymphocytes	-	**leukemia**, NHL, reticulosarcoma
chlorambucil (2708)	chlorambucil	Known	M	alkylating agent	**CLL**, HD, GN, KS, NHL, RA, WG, chronic hepatitis, systemic lupus, psoriasis, primary macroglobulinemia, cold agglutinin disease, nephrotic syndrome, and cancer of the breast, lung, cervix, ovaries, and testes	**AML**
cyclophosphamide (2907)	cyclophosphamide	Known	M	-	**leukemia,** MM, NB, RA, WG, lymphoma, mycosis fungoides, ovarian cancer, breast cancer, nephrotic syndrome in children	**leukemia,** bladder cancer
ethylene oxide (6354)	ethylene oxide	Known	E/I	an alkylating agent that forms DNA adduct	-	**lymphocytic leukemia,** NHL, reticulosarcoma, stomach cancer, breast cancer
formaldehyde (712)	formaldehyde	Known	E/I	forms reactive methanediol, which directly damages bone marrow and hematopoietic progenitors	-	**CML**, NPC, sinonasal cancer

Chemicals are ordered according to the cancers and diseases they are reported to cause, then by IARC group numbers {1, 2a, 2b, 3, then 4}, then alphabetically by CTD chemical name. Exposure types are expressed as E/I (Environmental or Industrial) or M (Medical/Therapeutic). Chemicals in *gray-italics* did not have associated human CTD data and were excluded from further data analysis.

Table S1b. Chemicals found to cause leukemia in humans, according to IARC Monographs.

		IARC monograph chemical summaries							NTP RoC chemical summaries	
CTD chemical name (Compound CID, if used)	CASRN	Chemical name	Group	Volume	Exposure type	Mode of action	Treatment use	Resultant diseases	Chemical name	Class
Reported to cause predominantly leukemas										
busulfan (2478)	55-98-1	busulfan	1	4, Sup7, 100A	M	a bifunctional, alkylating agent	**CML**, brochial carcinoma, thrombocytosis, polycythemia vera	**AML**, MDS, pancytopenia	1,4-butanediol dimethanesulfonate	Known
etoposide (36462)	33419-42-0	etoposide	1	76, 100A	M	inhibits DNA synthesis by interfering with the activity of DNA topoisomerase II	**AML, ALL, HD, NHL,** lung cancer, testicular neoplasms	**AML,** MDS	-	-
teniposide (452548)	29767-20-2	teniposide	2a	76	M	inhibits DNA topoisomerase II	**ALL**, **AML**, Childhood NB, lymphoma, adult brain tumors, bladder cancer, lung cancer	**AML** specifically in childhood cancers	-	-
treosulfan	*299-75-2*	*treosulfan*	*1*	*26, Sup 7, 100A*	*M*	*a bifunctional, alkylating agent that forms DNA crosslinks*	*ovarian cancer, breast cancer, melanoma*	*AML*	-	-
Reported to cause leukemias and other cancers										
trichloroethylene (6575)	79-01-6	trichloroethylene	2a	63	E/I	-	-	**leukemia, NHL**, liver cancer, biliary tract cancers, cervical cancer	trichloroethylene	Anticipated

Chemicals are ordered according to the cancers and diseases they are reported to cause, then by IARC group numbers {1, 2a, 2b, 3, then 4}, then alphabetically by CTD chemical name. Exposure types are expressed as E/I (Environmental or Industrial) or M (Medical/Therapeutic). Chemicals in gray-italics did not have associated human CTD data and were excluded from further data analysis. The NTP column is shortened to show only the source and class information, and grayed due to the absence of associations with human leukemias.

Table S1c. Chemicals found to cause leukemia in humans, according to NTP 12th Report on Carcinogens

CTD chemical name (Compound CID, if used)	CASRN
Reported to cause predominantly leukemias	
bcnu (2578)	154-93-8
cisplatin (84691)	15663-27-1
ccnu (3950)	13010-47-4
propylthiouracil (657298)	51-52-5
Reported to cause leukemias and other cancers	
vinyl chloride (6338)	75-01-4
adriamycin (31703)	23214-92-8
3,3'-dichlorobenzidine (7070)	91-94-1
DTT (3036)	50-29-3
lindane (727); alpha-hexachlorocyclohexane (727); beta-hexachlorocyclohexane (727); delta-hexachlorocyclohexane (727)	58-89-9; 319-84-6; 319-85-7; 319-86-8
nitrogen mustard (4033)	55-86-7; 51-75-2
oxymetholone (5281034)	434-07-1
styrene (7501)	100-42-5
thorium dioxide	*1314-20-1*
chloroprene	*126-99-8*
1,3-dichloropropene	*542-75-6*
phenoxybenzamine hydrochloride	*59-96-1; 63-92-3*

NTP RoC chemical summaries					
Chemical name	Class	Exposure type	Mode of action	Treatment use	Resultant diseases
Bis(chloroethyl) Nitrosourea	Anticipated	M	a bifunctionally alkylating agent	HD, MM, NHL, primary/metastatic brain tumors, burkitt's lymphoma, melanoma, Ewing's sarcoma, mycosis fungoides, breast cancer, gastrointestinal cancers	**AML**
cisplatin	Anticipated	M	forms cisplatin-induced platinum-DNA-adducts	ovarian cancer, testicular cancer	**secondary leukemia**
1-(2-chloroethyl)-3-cyclohexyl-1-nitrosourea	Anticipated	M	a bifunctional alkylating agent	brain tumors, HD, NHL, psoriasis, melanoma, mycosis fungoides, and cancer of the gastrointestinal tract, kidney, and breast	**leukemia**
propylthiouracil	Anticipated	M	an antithyroid agent	hyperthyroidism	**AML**
vinyl chloride	Known	E/I	the metabolite forms DNA adducts	-	**leukemia**, lymphoma, lung cancer, hepatic angiosarcoma, brain cancer, hepatocellular carcinoma
adriamycin	Anticipated	M	a cytotoxic anthracycline-class antibiotic used as an antimitotic	**acute leukemia**, HD, KS, MM, NB, NHL, OS, STS, Ewing's sarcoma, Wilms' tumor, and carcinoma of the head and neck, breast, thyroid gland, genitourinary tract, and lung	**AML**, OS
3,3'-dichlorobenzidine and its dihydrochloride	Anticipated	E/I	-	-	**leukemia**, kidney cancer
dichlorodiphenyl-trichloroethane	Anticipated	E/I	-	-	**leukemia**, MM, breast cancer
lindane, hexachlorocyclohexane (technical grade), and other hexachlorocyclohexane isomers	Anticipated	E/I	-	-	**paramyeloblastic leukemia**, **myelomonocytic leukemia**, lung cancer
Nitrogen mustard hydrochloride	Anticipated	M	-	**leukemia**, HD, NHL, polycythemia vera, mycosis fungoides, bronchogenic carcinoma, melanoma, and cancer of the kidney, gastrointestinal tract and breast	**leukemia**, SCC
oxymetholone	Anticipated	M	a synthetic androgen	AA, fanconi's anemia, paroxysmal nocturnal hemoglobinuria	**AML**, SCC of the head and neck, and cancer of the esophagus, liver, anogenital region, and bile-duct
styrene	Anticipated	E/I	-	-	**leukemia**, lymphoma, hematopoietic cancer
thorium dioxide	*Known*	*M*	*-*	*Used in medical radiology as an intravascular contrast agent for cerebral limb angiography*	*leukemia, hemangiosarcoma, bone cancers, primarily cholangiocellular and tumors*
chloroprene	*Anticipated*	*E/I*	*-*	*-*	*leukemia and other cancers of the liver, lung, digestive, hematopoietic system*
1,3-dichloropropene (technical grade)	*Anticipated*	*E/I*	*-*	*-*	*leukemia, pancreatic cancer, histiocytic lymphoma*
phenoxybenzamine hydrochloride	*Anticipated*	*M*	*an α-adrenergic receptor blocking agent used to treat peripheral vascular disorders*	*Raynaud's disease, phlebothrombosis, causalgia, diabetic gangrene, phlebitis, chronic skin ulcers, urinary-bladder problems, and sweating caused by pheochromocytoma*	*CLL, SCC, small-cell carcinoma, bladder cancer, esophageal cancer*

IARC monograph chemical summaries		
Chemical name	Group	Volume
Bischloroethyl nitrosourea (BCNU)	2A	26, Sup 7
cisplatin	2A	26, Sup 7
1-(2-Chloroethyl)-3-cyclohexyl-1-nitrosourea (CCNU)	2A	26, Sup 7
propylthiouracil	2B	79
vinyl chloride	1	97, 100F
adriamycin	2A	10, Sup 7
3,3'-dichlorobenzidine	2B	29, Sup 7
DDT (4,4'-Dichlorodiphenyl trichloroethane)	2B	53
hexachlorocyclohexanes	2B	20, Sup 7
nitrogen mustard	2A	9, Sup 7
-	-	-
styrene	2B	60, 82
-	-	-
chloroprene	*2B*	*71*
1,3-dichloropropene (technical-grade)	*2B*	*41, Sup 7, 71*
phenoxybenzamine hydrochloride	*2B*	*24, Sup 7*

Chemicals are ordered according to the cancers and diseases they are reported to cause, then by their "Known" or "Anticipated" NTP Report on Carcinogens (RoC) class, then alphabetically by CTD chemical name. Exposure types are expressed as E/I (Environmental or Industrial exposure) or M (Medical/Therapeutic exposure). Chemicals in *gray italics* did not have associated human CTD data and were excluded from further data analysis. The IARC column is shortened to show only the source and class information, if available, and grayed due to the absence of associations with human leukemias.

Table S2a. Chemicals identified as non-leukemogenic carcinogens, according to both the IARC monographs and the NTP 12th Report on Carcinogens.

		IARC monograph chemical summaries							NTP RoC chemical summaries					
CTD chemical name (Compound CID, if used)	CASRN	Chemical name	Group	Volume	Exposure type	Mode of action	Treatment use	Resultant diseases	Chemical name	Class	Exposure type	Mode of action	Treatment use	Resultant diseases
Reported to cause lymphomas														
azathioprine (2265)	446-86-6	Azathioprine	1	26, Sup 7, 100A	M	induces DNA damage by 6-thioguanine accumulation in the DNA of patients	RA, renal-transplant allograft rejection preventative, systemic lupus, inflammatory bowel disease	NHL, SCC, mesenchymal tumors, hepatobiliary carcinoma	Azathioprine	Known	M	-	kidney-transplant rejection preventative, systemic lupus, Crohn's disease, autoimmune hemolytic anemia, myasthenia gravis, chronic hepatitis, ulcerative colitis	NHL, SCC, mesenchymal tumor, hepatobiliary carcinoma
cyclosporine (2907)	59865-13-3	Cyclosporine	1	50, 100A	M	-	allograft rejection preventative, systemic lupus, dermatomyositis, Grave's disease, Crohn's disease, myasthenia gravis, chronic hepatitis, biliary cirrhosis, uveitis, sarcoidosis, psoriasis	KS, lymphoma, skin cancer	Cyclosporin A	Known	M	-	RA, psoriasis	KS, skin cancer, lymphoma
TCDD (15625)	1746-1-6	2,3,7,8-Tetrachloro dibenzo-para-dioxin	1	69, 100F	E/I	sustained AHR binding and anti-apoptosis gene expression	-	NHL, STS, lung cancer	2,3,7,8-Tetrachloro dibenzo-para-dioxin	Known	E/I	-	-	NHL, lung cancer
tetrachloroethylene (31373)	127-18-4	Tetrachloro-ethylene	2a	63	E/I	-	-	NHL, esophageal cancer, cervical cancer, and a potential risk of leukemia	Tetrachloro-ethylene	Anticipated	E/I	-	-	NHL, esophageal cancer, cervical cancer
Reported to cause primarily lung and respiratory cancers														
sulfur mustard (10461)	505-60-2	Sulfur mustard	1	9, Sup 7, 100F	E/I	-	-	lung cancer, larynx cancer	Mustard gas	Known	E/I	causes genetic damage chromosome aberrations	-	cancer of the lung, and larynx, pharynx, and upper-respiratory tract
bis(chloromethyl) ether	*542-88-1*	*Bis(chloromethyl) Ether*	*1*	*4, Sup7, 100F*	*E/I*	*an alkylating agent*	*-*	*lung cancer*	*Bis(chloromethyl) ether*	*Known*	*E/I*	*an alkylating agent that induces unschedule DNA synthesis and forms chromosome aberrations in white blood cells*	*-*	*lung cancer*
chloromethyl methyl ether	*107-30-2*	*Chloromethyl methyl ether*	*1*	*4, Sup7, 100F*	*E/I*	*an alkylating agent*	*-*	*lung cancer*	*Technical-Grade Chloromethyl Methyl Ether*	*Known*	*E/I*	*an alkylating agent forms chromosome aberrations in white blood cells*	*-*	*lung cancer*
Reported to cause primarily bladder, liver kidney, and gastrointestinal cancers														
4-aminobiphenyl (7102)	92-67-1	4-aminobiphenyl	1	1, Sup7, 99, 100F	E/I	-	-	bladder cancer	4-aminobiphenyl	Known	E/I	-	-	bladder cancer, liver cancer
benzidine (7111)	92-87-5	Benzidine	1	29, Sup7, 99, 100F	E/I	the metabolite forms DNA adducts to induce clastogenic effects	-	bladder cancer	Benzidine	Known	E/I	the metabolite binds DNA and forms chromosome aberrations in the white blood cells	-	bladder cancer
2-naphthylamine (7057)	91-59-8	2-Naphthylamine	1	4, Sup 7, 99, 100F	E/I	the metabolite forms DNA adduct	-	bladder cancer	2-Naphthylamine	Known	E/I	the metabolite likely forms adducts with blood-serum proteins	-	bladder cancer
phenacetin (4754)	62-44-2	Phenacetin	1	24, Sup 7, 100A	E/I	-	fever reducer, pain reliever	kidney cancer, ureter cancer	Phenacetin	Anticipated	M	-	pain, fever	TCC of the renal pelvis
aristolochic acid	*313-67-7*	*Plants containing Aristolochic acid*	*1*	*82, M 100A*	*-*	*inflammation, edema*		*human nephropathy, urothelial cancer*	*Aristolochic acid*	*Known*	*M*	*a relatively selective inhibitor of phospholipase A2*	*antibacterial, antiviral, antifungal, antitumor, inflammatory diseases, venomous infections*	*urothelial cancer, aristolochic acid nephropathy, progressive interstitial renal fibrosis*
2-toluidine	*95-53-4*	*ortho-Toluidine*	*1*	*77, 99, 100F*	*E/I*	*the metabolite forms DNA adduct*	*-*	*bladder cancer*	*ortho-Toluidine*	*Anticipated*	*E/I*	*-*	*-*	*bladder cancer*

Table S2a. *Cont.*

CTD chemical name (Compound CID, if used) CASRN	IARC monograph chemical summaries: Chemical name	Group	Volume	Exposure type	Mode of action	Treatment use	Resultant diseases	NTP RoC chemical summaries: Chemical name	Class	Exposure type	Mode of action	Treatment use	Resultant diseases
Reported to cause primarily reproductive orang cancers													
diethylstilbestrol (448537) 56-53-1	Diethylstilbestrol	1	21, Sup 7, 100A	E/I	-	menopausal symptoms, senile vaginitis, vulvar dystrophy	clear-cell adenocarcinoma of the vagina/cervix, testicular cancer, breast cancer, endometrial cancer	Diethylstilbestrol	Known	M	a synthetic estrogen	prostate cancer, breast cancer in women (postmenopausal)	clear-cell adenocarcinoma of the vagina/cervix, breast cancer, testicular cancer
tamoxifen (2733526) 10540-29-1	Tamoxifen	1	66, 100A	M	-	metastatic breast cancer	endometrium cancer	Tamoxifen	Known	M	-	contralateral breast cancer	endometrial uterine cancer

Chemicals are ordered according to the cancers and diseases they are reported to cause, then by their IARC Group numbers {1, 2a, 2b, 3, 4}, then alphabetically by CTD chemical name. Exposure types are indicated as E/I (Environmental or Industrial) or M (Medical/Therapeutic). Chemicals in *gray italics* did not have associated human CTD data and were excluded from further data analysis.

Table S2b. Chemicals identified as non-leukemogenic carcinogens, according to the IARC monographs.

CTD chemical name (Compound CID, if used) CASRN	IARC monograph chemical summaries: Chemical name	Group	Volume	Exposure type	Mode of action	Treatment use	Resultant diseases	NTP RoC chemical summaries: Chemical name	Class
Reported to cause bladder, liver, kidney, and gastrointestinal cancers									
chlornaphazine 494-03-1	*Chlornaphazine*	*1*	*4, Sup7, 100A*	*M*	*a bifunctional alkylating agent*	*HD, polycythemia vera*	*invasive carcinoma of the bladder, papillary carcinoma grade II of the bladder*	-	-

Chemical names in *gray italics* did not have associated human CTD data and were excluded from further data analysis. The NTP data is truncated to show only the source and class information. Exposure type is indicated as M for a Medical/Therapeutic.

Table S3. Definitions of disease abbreviations used in Tables S1 and S2.

Abbreviations	Full name of abbreviated diseases
AA	Aplastic Anemia
AML	Acute Myeloid Leukemia (also Acute Non-lymphocytic Leukemia)
ALL	Acute Lymphoblastic Leukemia (also Acute Lymphocytic Leukemia)
CLL	Chronic Lymphocytic Leukemia
CML	Chronic Myeloid Leukemia (also Chronic Non-lymphocytic Leukemia)
CTCL	Cutaneous T-cell Lymphoma
GN	Glomerulonephritis (also Glomerular nephritis)
HD	Hodgkin's Disease (also Hodgkin's Lymphoma)
KS	Kaposi's Sarcoma (also Kaposi's Disease)
MM	Multiple Myeloma
MDS	Myelodysplastic Syndrome
NB	Neuroblastoma
NHL	Non-Hodgkin's Lymphoma (also Lymphosarcoma)
NPC	Nasopharyngeal Cancer
OS	Osteogenic Sarcoma (also Osteosarcoma)
RA	Rheumatoid Arthritis
SCC	Squamous Cell Carcinoma
STS	Soft-Tissue Sarcoma (also rhabdomyosarcoma)
TCC	Transitional Cell Carcinoma
WM	Waldenström macroglobulinemia (also Lymphoplasmacytic Lymphoma)
WG	Wegener's granulomatosis

Table S4. Number of leukemogens and non-leukemogenic carcinogens targeting each of the 250 KEGG human pathways; Membership probabilities of each pathway in the two clusters in Figure 1; and, the Mean decrease in gini index scores from the random forest classification method.

KEGG ID	All Pathways	Disease / Biological Pathway*	Leukemogens (n = 29)		Non- Leukemogenic Carcinogens (n = 11)		Cluster 0 Probability	Cluster 1 Probability	Mean Decrease Gini
			No.	%	No.	%			
path:hsa00010	Glycolysis / Gluconeogenesis	0	8	28	4	36	0.68	0.33	0.02
path:hsa00020	Citrate cycle (TCA cycle)	0	1	3	1	9	0.99	0.01	0.03
path:hsa00030	Pentose phosphate pathway	0	3	10	2	18	0.98	0.02	0.02
path:hsa00040	Pentose and glucuronate interconversions	0	7	24	5	45	0.89	0.11	0.14
path:hsa00051	Fructose and mannose metabolism	0	0	0	2	18	1.00	0.00	0.01
path:hsa00052	Galactose metabolism	0	3	10	3	27	0.98	0.02	0.07
path:hsa00053	Ascorbate and aldarate metabolism	0	6	21	4	36	0.87	0.13	0.09
path:hsa00061	Fatty acid biosynthesis	0	2	7	1	9	0.95	0.05	0.00
path:hsa00062	Fatty acid elongation	0	5	17	2	18	0.82	0.18	0.01
path:hsa00071	Fatty acid metabolism	0	6	21	2	18	0.93	0.07	0.02
path:hsa00072	Synthesis and degradation of ketone bodies	0	1	3	2	18	0.85	0.15	0.02
path:hsa00100	Steroid biosynthesis	0	3	10	2	18	0.88	0.12	0.03
path:hsa00120	Primary bile acid biosynthesis	0	0	0	3	27	1.00	0.00	0.11
path:hsa00130	Ubiquinone and other terpenoid-quinone biosynthesis	0	0	0	2	18	1.00	0.00	0.03
path:hsa00140	Steroid hormone biosynthesis	0	14	48	5	45	0.03	0.97	0.09
path:hsa00190	Oxidative phosphorylation	0	0	0	1	9	0.64	0.36	0.04
path:hsa00230	Purine metabolism	0	7	24	3	27	0.01	0.99	0.04
path:hsa00232	Caffeine metabolism	0	3	10	8	73	0.30	0.70	0.36
path:hsa00240	Pyrimidine metabolism	0	7	24	0	0	0.66	0.35	0.05
path:hsa00250	Alanine, aspartate and glutamate metabolism	0	3	10	3	27	0.95	0.05	0.03

Table S4. *Cont.*

KEGG ID	All Pathways	Disease / Biological Pathway*	Leukemogens (n = 29)		Non- Leukemogenic Carcinogens (n = 11)		Cluster 0 Probability	Cluster 1 Probability	Mean Decrease Gini
			No.	%	No.	%			
path:hsa00260	Glycine, serine and threonine metabolism	0	6	21	3	27	0.86	0.14	0.04
path:hsa00270	Cysteine and methionine metabolism	0	4	14	1	9	0.81	0.19	0.03
path:hsa00280	Valine, leucine and isoleucine degradation	0	4	14	2	18	0.96	0.04	0.02
path:hsa00290	Valine, leucine and isoleucine biosynthesis	0	0	0	2	18	1.00	0.00	0.02
path:hsa00300	Lysine biosynthesis	0	1	3	1	9	1.00	0.00	0.00
path:hsa00310	Lysine degradation	0	7	24	2	18	0.82	0.18	0.02
path:hsa00330	Arginine and proline metabolism	0	6	21	5	45	0.37	0.63	0.07
path:hsa00340	Histidine metabolism	0	5	17	2	18	0.61	0.39	0.02
path:hsa00350	Tyrosine metabolism	0	7	24	3	27	0.24	0.77	0.03
path:hsa00360	Phenylalanine metabolism	0	4	14	3	27	0.58	0.42	0.06
path:hsa00380	Tryptophan metabolism	0	10	34	7	64	0.02	0.98	0.12
path:hsa00400	Phenylalanine, tyrosine and tryptophan biosynthesis	0	0	0	0	0	1.00	0.00	0.02
path:hsa00410	beta-Alanine metabolism	0	8	28	3	27	0.79	0.21	0.02
path:hsa00430	Taurine and hypotaurine metabolism	0	0	0	2	18	1.00	0.00	0.04
path:hsa00450	Selenocompound metabolism	0	6	21	2	18	0.91	0.09	0.01
path:hsa00460	Cyanoamino acid metabolism	0	0	0	2	18	0.91	0.09	0.01
path:hsa00471	D-Glutamine and D-glutamate metabolism	0	3	10	2	18	0.93	0.07	0.03
path:hsa00472	D-Arginine and D-ornithine metabolism	0	0	0	2	18	1.00	0.00	0.03
path:hsa00480	Glutathione metabolism	0	18	62	3	27	0.02	0.98	0.10
path:hsa00500	Starch and sucrose metabolism	0	1	3	4	36	0.84	0.16	0.10

Table S4. *Cont.*

KEGG ID	All Pathways	Disease / Biological Pathway*	Leukemogens (n = 29)		Non- Leukemogenic Carcinogens (n = 11)		Cluster 0 Probability	Cluster 1 Probability	Mean Decrease Gini
			No.	%	No.	%			
path:hsa00510	N-Glycan biosynthesis	0	3	10	3	27	0.87	0.14	0.02
path:hsa00511	Other glycan degradation	0	1	3	1	9	0.96	0.04	0.03
path:hsa00512	Mucin type O-Glycan biosynthesis	0	3	10	3	27	0.93	0.07	0.05
path:hsa00514	Other types of O-glycan biosynthesis	0	4	14	3	27	0.77	0.23	0.13
path:hsa00520	Amino sugar and nucleotide sugar metabolism	0	2	7	3	27	0.84	0.16	0.09
path:hsa00524	Butirosin and neomycin biosynthesis	0	1	3	2	18	1.00	0.00	0.04
path:hsa00531	Glycosaminoglycan degradation	0	2	7	2	18	0.98	0.03	0.01
path:hsa00532	Glycosaminoglycan biosynthesis—chondroitin sulfate	0	4	14	3	27	0.62	0.38	0.01
path:hsa00533	Glycosaminoglycan biosynthesis—keratan sulfate	0	2	7	2	18	1.00	0.00	0.01
path:hsa00534	Glycosaminoglycan biosynthesis—heparan sulfate	0	3	10	2	18	0.97	0.03	0.02
path:hsa00561	Glycerolipid metabolism	0	5	17	2	18	0.9	0.11	0.01
path:hsa00562	Inositol phosphate metabolism	0	2	7	3	27	0.9	0.10	0.05
path:hsa00563	Glycosylphosphatidylinositol(GPI)-anchor biosynthesis	0	2	7	1	9	0.77	0.23	0.02
path:hsa00564	Glycerophospholipid metabolism	0	2	7	4	36	0.67	0.33	0.07
path:hsa00565	Ether lipid metabolism	0	1	3	3	27	0.91	0.09	0.08
path:hsa00590	Arachidonic acid metabolism	0	13	45	6	55	0.03	0.97	0.20
path:hsa00591	Linoleic acid metabolism	0	10	34	4	36	0.01	0.99	0.10
path:hsa00592	Alpha-Linolenic acid metabolism	0	0	0	2	18	1.00	0.00	0.04
path:hsa00600	Sphingolipid metabolism	0	6	21	3	27	0.96	0.04	0.03
path:hsa00601	Glycosphingolipid biosynthesis—lacto and neolacto series	0	2	7	3	27	0.98	0.02	0.03

Table S4. *Cont.*

KEGG ID	All Pathways	Disease / Biological Pathway*	Leukemogens (n = 29)		Non- Leukemogenic Carcinogens (n = 11)		Cluster 0 Probability	Cluster 1 Probability	Mean Decrease Gini
			No.	%	No.	%			
path:hsa00603	Glycosphingolipid biosynthesis—globo series	0	1	3	1	9	0.96	0.04	0.04
path:hsa00604	Glycosphingolipid biosynthesis—ganglio series	0	3	10	2	18	0.99	0.01	0.03
path:hsa00620	Pyruvate metabolism	0	5	17	2	18	0.86	0.14	0.01
path:hsa00630	Glyoxylate and dicarboxylate metabolism	0	1	3	2	18	0.99	0.01	0.02
path:hsa00640	Propanoate metabolism	0	5	17	2	18	0.78	0.23	0.01
path:hsa00650	Butanoate metabolism	0	3	10	2	18	0.87	0.13	0.02
path:hsa00670	One carbon pool by folate	0	4	14	2	18	0.95	0.05	0.02
path:hsa00730	Thiamine metabolism	0	0	0	2	18	1.00	0.00	0.02
path:hsa00740	Riboflavin metabolism	0	2	7	2	18	1.00	0.00	0.04
path:hsa00750	Vitamin B6 metabolism	0	2	7	2	18	1.00	0.00	0.01
path:hsa00760	Nicotinate and nicotinamide metabolism	0	3	10	3	27	0.89	0.11	0.02
path:hsa00770	Pantothenate and CoA biosynthesis	0	2	7	2	18	0.96	0.04	0.01
path:hsa00780	Biotin metabolism	0	0	0	1	9	1.00	0.00	0.00
path:hsa00785	Lipoic acid metabolism	0	1	3	0	0	0.36	0.64	0.00
path:hsa00790	Folate biosynthesis	0	3	10	4	36	0.82	0.18	0.04
path:hsa00830	Retinol metabolism	0	15	52	8	73	0.02	0.98	0.15
path:hsa00860	Porphyrin and chlorophyll metabolism	0	3	10	2	18	0.47	0.53	0.11
path:hsa00900	Terpenoid backbone biosynthesis	0	1	3	2	18	0.97	0.04	0.02
path:hsa00910	Nitrogen metabolism	0	8	28	3	27	0.49	0.51	0.02
path:hsa00920	Sulfur metabolism	0	1	3	4	36	0.47	0.53	0.11
path:hsa00970	Aminoacyl-tRNA biosynthesis	0	2	7	2	18	0.78	0.22	0.05
path:hsa00980	Metabolism of xenobiotics by cytochrome P450	0	20	69	7	64	0.00	1.00	0.15

Table S4. *Cont.*

KEGG ID	All Pathways	Disease / Biological Pathway*	Leukemogens (n = 29)		Non- Leukemogenic Carcinogens (n = 11)		Cluster 0 Probability	Cluster 1 Probability	Mean Decrease Gini
			No.	%	No.	%			
path:hsa00982	Drug metabolism—cytochrome P450	0	13	45	5	45	0.01	0.99	0.11
path:hsa00983	Drug metabolism—other enzymes	0	9	31	5	45	0.00	1.00	0.16
path:hsa01040	Biosynthesis of unsaturated fatty acids	0	4	14	2	18	0.64	0.36	0.02
path:hsa01100	Metabolic pathways	0	12	41	5	45	0.00	1.00	0.09
path:hsa02010	ABC transporters	0	7	24	1	9	0.27	0.73	0.04
path:hsa03008	Ribosome biogenesis in eukaryotes	0	3	10	2	18	0.64	0.36	0.03
path:hsa03010	Ribosome	0	2	7	5	45	0.62	0.38	0.15
path:hsa03013	RNA transport	0	5	17	2	18	0.01	0.99	0.04
path:hsa03015	mRNA surveillance pathway	0	3	10	4	36	0.67	0.33	0.03
path:hsa03018	RNA degradation	0	5	17	3	27	0.82	0.18	0.03
path:hsa03020	RNA polymerase	0	2	7	0	0	0.79	0.21	0.01
path:hsa03022	Basal transcription factors	0	5	17	3	27	0.76	0.24	0.02
path:hsa03030	DNA replication	0	1	3	3	27	1.00	0.00	0.06
path:hsa03040	Spliceosome	0	9	31	0	0	0.63	0.38	0.02
path:hsa03050	Proteasome	0	4	14	6	55	0.57	0.43	0.07
path:hsa03060	Protein export	0	3	10	1	9	0.36	0.64	0.02
path:hsa03320	PPAR signaling pathway	0	7	24	4	36	0.1	0.90	0.02
path:hsa03410	Base excision repair	0	11	38	2	18	0.02	0.98	0.03
path:hsa03420	Nucleotide excision repair	0	4	14	4	36	0.59	0.41	0.02
path:hsa03430	Mismatch repair	0	3	10	3	27	0.82	0.18	0.04
path:hsa03440	Homologous recombination	0	8	28	4	36	0.19	0.81	0.03
path:hsa03450	Non-homologous end-joining	0	3	10	1	9	0.81	0.19	0.02
path:hsa03460	Fanconi anemia pathway	0	7	24	4	36	0.18	0.82	0.03
path:hsa04010	MAPK signaling pathway	0	17	59	7	64	0.00	1.00	0.11
path:hsa04012	ErbB signaling pathway	0	15	52	4	36	0.00	1.00	0.07
path:hsa04020	Calcium signaling pathway	0	4	14	4	36	0.00	1.00	0.06

Table S4. *Cont.*

KEGG ID	All Pathways	Disease / Biological Pathway*	Leukemogens (n = 29)		Non- Leukemogenic Carcinogens (n = 11)		Cluster 0 Probability	Cluster 1 Probability	Mean Decrease Gini
			No.	%	No.	%			
path:hsa04060	Cytokine-cytokine receptor interaction	0	12	41	6	55	0.00	1.00	0.07
path:hsa04062	Chemokine signaling pathway	0	10	34	4	36	0.00	1.00	0.04
path:hsa04070	Phosphatidylinositol signaling system	0	3	10	2	18	0.63	0.37	0.08
path:hsa04080	Neuroactive ligand-receptor interaction	0	8	28	4	36	0.00	1.00	0.03
path:hsa04110	Cell cycle	0	13	45	7	64	0.21	0.80	0.09
path:hsa04114	Oocyte meiosis	0	12	41	3	27	0.01	0.99	0.04
path:hsa04115	p53 signaling pathway	0	16	55	8	73	0.11	0.89	0.06
path:hsa04120	Ubiquitin mediated proteolysis	0	3	10	2	18	0.62	0.38	0.02
path:hsa04122	Sulfur relay system	0	1	3	0	0	0.75	0.25	0.00
path:hsa04130	SNARE interactions in vesicular transport	0	5	17	3	27	0.75	0.25	0.02
path:hsa04140	Regulation of autophagy	0	6	21	3	27	0.37	0.63	0.04
path:hsa04141	Protein processing in endoplasmic reticulum	0	11	38	3	27	0.00	1.00	0.05
path:hsa04142	Lysosome	0	2	7	3	27	0.63	0.38	0.09
path:hsa04144	Endocytosis	0	10	34	3	27	0.00	1.00	0.03
path:hsa04145	Phagosome	0	6	21	6	55	0.01	1.00	0.07
path:hsa04146	Peroxisome	0	7	24	2	18	0.54	0.46	0.04
path:hsa04150	mTOR signaling pathway	0	11	38	2	18	0.00	1.00	0.04
path:hsa04210	Apoptosis	0	18	62	9	82	0.01	0.99	0.08
path:hsa04260	Cardiac muscle contraction	0	2	7	0	0	0.61	0.39	0.02
path:hsa04270	Vascular smooth muscle contraction	0	12	41	6	55	0.05	0.95	0.04
path:hsa04270	Vascular smooth muscle contraction	0	12	41	6	55	0.05	0.95	0.04
path:hsa04310	Wnt signaling pathway	0	10	34	5	45	0.00	1.00	0.10
path:hsa04320	Dorso-ventral axis formation	0	8	28	3	27	0.01	0.99	0.03

Table S4. *Cont.*

KEGG ID	All Pathways	Disease / Biological Pathway*	Leukemogens (n = 29)		Non- Leukemogenic Carcinogens (n = 11)		Cluster 0 Probability	Cluster 1 Probability	Mean Decrease Gini
			No.	%	No.	%			
path:hsa04330	Notch signaling pathway	0	4	14	2	18	0.92	0.08	0.02
path:hsa04340	Hedgehog signaling pathway	0	3	10	3	27	0.95	0.05	0.06
path:hsa04350	TGF-beta signaling pathway	0	13	45	7	64	0.05	0.95	0.08
path:hsa04360	Axon guidance	0	9	31	3	27	0.01	0.99	0.04
path:hsa04370	VEGF signaling pathway	0	12	41	7	64	0.00	1.00	0.08
path:hsa04380	Osteoclast differentiation	0	12	41	4	36	0.00	1.00	0.05
path:hsa04510	Focal adhesion	0	12	41	5	45	0.00	1.00	0.08
path:hsa04512	ECM-receptor interaction	0	7	24	4	36	0.34	0.66	0.10
path:hsa04514	Cell adhesion molecules (CAMs)	0	4	14	4	36	0.65	0.35	0.05
path:hsa04520	Adherens junction	0	10	34	2	18	0.16	0.84	0.05
path:hsa04530	Tight junction	0	4	14	1	9	0.67	0.33	0.02
path:hsa04540	Gap junction	0	14	48	4	36	0.00	1.00	0.05
path:hsa04610	Complement and coagulation cascades	0	2	7	2	18	0.55	0.46	0.04
path:hsa04612	Antigen processing and presentation	0	8	28	7	64	0.02	0.98	0.09
path:hsa04614	Renin-angiotensin system	0	2	7	3	27	0.37	0.63	0.13
path:hsa04620	Toll-like receptor signaling pathway	0	17	59	7	64	0.00	1.00	0.06
path:hsa04621	NOD-like receptor signaling pathway	0	14	48	5	45	0.00	1.00	0.10
path:hsa04622	RIG-I-like receptor signaling pathway	0	9	31	5	45	0.01	0.99	0.10
path:hsa04623	Cytosolic DNA-sensing pathway	0	9	31	4	36	0.02	0.98	0.03
path:hsa04630	Jak-STAT signaling pathway	0	11	38	7	64	0.00	1.00	0.07
path:hsa04640	Hematopoietic cell lineage	0	10	34	4	36	0.00	1.00	0.05
path:hsa04650	Natural killer cell mediated cytotoxicity	0	13	45	7	64	0.00	1.00	0.05
path:hsa04660	T cell receptor signaling pathway	0	12	41	4	36	0.00	1.00	0.06
path:hsa04662	B cell receptor signaling pathway	0	13	45	4	36	0.01	1.00	0.05
path:hsa04664	Fc epsilon RI signaling pathway	0	9	31	6	55	0.00	1.00	0.05

Table S4. *Cont.*

**KEGG ID**	**All Pathways**	**Disease / Biological Pathway***	**Leukemogens (n = 29)**		**Non- Leukemogenic Carcinogens (n = 11)**		**Cluster 0 Probability**	**Cluster 1 Probability**	**Mean Decrease Gini**
			**No.**	**%**	**No.**	**%**			
path:hsa04666	Fc gamma R-mediated phagocytosis	0	10	34	3	27	0.03	0.97	0.04
path:hsa04670	Leukocyte transendothelial migration	0	7	24	4	36	0.51	0.49	0.04
path:hsa04672	Intestinal immune network for IgA production	0	9	31	5	45	0.00	1.00	0.06
path:hsa04710	Circadian rhythm—mammal	0	2	7	2	18	0.99	0.01	0.01
path:hsa04720	Long—term potentiation	0	12	41	4	36	0.20	0.80	0.06
path:hsa04721	Synaptic vesicle cycle	0	1	3	1	9	0.70	0.30	0.01
path:hsa04722	Neurotrophin signaling pathway	0	19	66	10	91	0.00	1.00	0.10
path:hsa04723	Retrograde endocannabinoid signaling	0	7	24	2	18	0.01	0.99	0.07
path:hsa04724	Glutamatergic synapse	0	8	28	3	27	0.08	0.92	0.03
path:hsa04725	Cholinergic synapse	0	15	52	5	45	0.00	1.00	0.06
path:hsa04726	Serotonergic synapse	0	12	41	5	45	0.00	1.00	0.10
path:hsa04727	GABAergic synapse	0	6	21	1	9	0.25	0.75	0.02
path:hsa04728	Dopaminergic synapse	0	7	24	5	45	0.21	0.79	0.08
path:hsa04730	Long-term depression	0	8	28	2	18	0.09	0.91	0.04
path:hsa04740	Olfactory transduction	0	0	0	0	0	0.15	0.86	0.02
path:hsa04742	Taste transduction	0	3	10	3	27	1.00	0.00	0.02
path:hsa04744	Phototransduction	0	2	7	2	18	0.63	0.37	0.06
path:hsa04810	Regulation of actin cytoskeleton	0	12	41	4	36	0.00	1.00	0.04
path:hsa04910	Insulin signaling pathway	0	11	38	5	45	0.01	0.99	0.05
path:hsa04912	GnRH signaling pathway	0	12	41	4	36	0.00	1.00	0.05
path:hsa04914	Progesterone-mediated oocyte maturation	0	13	45	3	27	0.00	1.00	0.02
path:hsa04916	Melanogenesis	0	11	38	3	27	0.01	0.99	0.05
path:hsa04920	Adipocytokine signaling pathway	0	7	24	7	64	0.00	1.00	0.08
path:hsa04930	Type II diabetes mellitus	1	10	34	1	9	0.00	1.00	0.03

Table S4. *Cont.*

KEGG ID	All Pathways	Disease / Biological Pathway*	Leukemogens (n = 29)		Non- Leukemogenic Carcinogens (n = 11)		Cluster 0 Probability	Cluster 1 Probability	Mean Decrease Gini
			No.	%	No.	%			
path:hsa04940	Type I diabetes mellitus	1	9	31	7	64	0.00	1.00	0.09
path:hsa04950	Maturity onset diabetes of the young	0	2	7	1	9	0.98	0.02	0.01
path:hsa04960	Aldosterone-regulated sodium reabsorption	0	13	45	5	45	0.01	1.00	0.04
path:hsa04961	Endocrine and other factor-regulated calcium reabsorption	0	5	17	3	27	0.16	0.84	0.03
path:hsa04962	Vasopressin-regulated water reabsorption	0	3	10	3	27	0.67	0.33	0.09
path:hsa04964	Proximal tubule bicarbonate reclamation	0	4	14	2	18	0.72	0.28	0.04
path:hsa04966	Collecting duct acid secretion	0	2	7	2	18	1.00	0.00	0.07
path:hsa04970	Salivary secretion	0	4	14	4	36	0.64	0.36	0.13
path:hsa04971	Gastric acid secretion	0	5	17	4	36	0.47	0.53	0.07
path:hsa04972	Pancreatic secretion	0	2	7	2	18	0.64	0.36	0.02
path:hsa04973	Carbohydrate digestion and absorption	0	6	21	1	9	0.94	0.06	0.02
path:hsa04974	Protein digestion and absorption	0	4	14	3	27	0.74	0.26	0.02
path:hsa04975	Fat digestion and absorption	0	2	7	1	9	0.75	0.25	0.02
path:hsa04976	Bile secretion	0	15	52	4	36	0.05	0.95	0.07
path:hsa04977	Vitamin digestion and absorption	0	3	10	1	9	0.48	0.52	0.02
path:hsa04978	Mineral absorption	0	7	24	2	18	0.55	0.45	0.02
path:hsa05010	Alzheimer's disease	1	13	45	4	36	0.00	1.00	0.06
path:hsa05012	Parkinson's disease	1	7	24	5	45	0.16	0.84	0.05
path:hsa05014	Amyotrophic lateral sclerosis (ALS)	1	18	62	8	73	0.00	1.00	0.08
path:hsa05016	Huntington's disease	1	11	38	4	36	0.00	1.00	0.07
path:hsa05020	Prion diseases	1	14	48	6	55	0.00	1.00	0.07
path:hsa05030	Cocaine addiction	0	7	24	4	36	0.03	0.97	0.04

Table S4. *Cont.*

KEGG ID	All Pathways	Disease / Biological Pathway*	Leukemogens (n = 29)		Non- Leukemogenic Carcinogens (n = 11)		Cluster 0 Probability	Cluster 1 Probability	Mean Decrease Gini
			No.	%	No.	%			
path:hsa05031	Amphetamine addiction	0	7	24	4	36	0.06	0.94	0.05
path:hsa05100	Bacterial invasion of epithelial cells	0	4	14	4	36	0.65	0.35	0.13
path:hsa05110	Vibrio cholerae infection	0	5	17	2	18	0.67	0.33	0.02
path:hsa05120	Epithelial cell signaling in Helicobacter pylori infection	0	12	41	6	55	0.00	1.00	0.04
path:hsa05130	Pathogenic Escherichia coli infection	0	2	7	2	18	0.90	0.10	0.04
path:hsa05131	Shigellosis	1	13	45	5	45	0.01	1.00	0.05
path:hsa05132	Salmonella infection	1	12	41	5	45	0.00	1.00	0.05
path:hsa05133	Pertussis	1	13	45	6	55	0.00	1.00	0.08
path:hsa05134	Legionellosis	1	14	48	4	36	0.00	1.00	0.09
path:hsa05140	Leishmaniasis	1	10	34	8	73	0.00	1.00	0.11
path:hsa05142	Chagas disease (American trypanosomiasis)	1	14	48	6	55	0.00	1.00	0.08
path:hsa05143	African trypanosomiasis	0	8	28	6	55	0.00	1.00	0.08
path:hsa05144	Malaria	1	10	34	6	55	0.00	1.00	0.11
path:hsa05145	Toxoplasmosis	1	17	59	6	55	0.00	1.00	0.09
path:hsa05146	Amoebiasis	1	11	38	6	55	0.00	1.00	0.07
path:hsa05150	Staphylococcus aureus infection	0	3	10	2	18	0.31	0.69	0.05
path:hsa05152	Tuberculosis	1	16	55	7	64	0.00	1.00	0.06
path:hsa05160	Hepatitis C	0	12	41	6	55	0.00	1.00	0.08
path:hsa05162	Measles	1	14	48	7	64	0.00	1.00	0.11
path:hsa05164	Influenza A	1	13	45	6	55	0.00	1.00	0.07
path:hsa05166	HTLV-I infection	1	16	55	7	64	0.02	0.98	0.08
path:hsa05168	Herpes simplex infection	0	12	41	7	64	0.00	1.00	0.08
path:hsa05200	Pathways in cancer	1	23	79	6	55	0.00	1.00	0.10
path:hsa05202	Transcriptional misregulation in cancer	1	14	48	7	64	0.00	1.00	0.12

Table S4. *Cont.*

KEGG ID	All Pathways	Disease / Biological Pathway*	Leukemogens (n = 29)		Non- Leukemogenic Carcinogens (n = 11)		Cluster 0 Probability	Cluster 1 Probability	Mean Decrease Gini
			No.	%	No.	%			
path:hsa05210	Colorectal cancer	1	20	69	6	55	0.00	1.00	0.10
path:hsa05211	Renal cell carcinoma	0	8	28	4	36	0.00	1.00	0.04
path:hsa05212	Pancreatic cancer	1	18	62	8	73	0.09	0.91	0.11
path:hsa05213	Endometrial cancer	1	16	55	7	64	0.00	1.00	0.05
path:hsa05214	Glioma	1	15	52	6	55	0.00	1.00	0.09
path:hsa05215	Prostate cancer	1	20	69	7	64	0.14	0.86	0.14
path:hsa05216	Thyroid cancer	1	11	38	7	64	0.00	1.00	0.09
path:hsa05217	Basal cell carcinoma	1	9	31	6	55	0.12	0.88	0.18
path:hsa05218	Melanoma	1	19	66	7	64	0.00	1.00	0.08
path:hsa05219	Bladder cancer	1	19	66	6	55	0.10	0.91	0.08
path:hsa05220	Chronic myeloid leukemia	1	18	62	7	64	0.01	1.00	0.08
path:hsa05221	Acute myeloid leukemia	1	11	38	3	27	0.06	0.94	0.06
path:hsa05222	Small cell lung cancer	1	18	62	9	82	0.07	0.93	0.07
path:hsa05223	Non-small cell lung cancer	1	16	55	6	55	0.00	1.00	0.09
path:hsa05310	Asthma	1	7	24	4	36	0.00	1.00	0.06
path:hsa05320	Autoimmune thyroid disease	1	10	34	6	55	0.00	1.00	0.06
path:hsa05322	Systemic lupus erythematosus	1	8	28	5	45	0.37	0.64	0.10
path:hsa05323	Rheumatoid arthritis	1	11	38	6	55	0.00	1.00	0.07
path:hsa05330	Allograft rejection	0	8	28	5	45	0.00	1.00	0.07
path:hsa05332	Graft-versus-host disease	0	10	34	6	55	0.00	1.00	0.06
path:hsa05340	Primary immunodeficiency	0	2	7	4	36	0.55	0.45	0.13
path:hsa05410	Hypertrophic cardiomyopathy (HCM)	1	11	38	5	45	0.01	0.99	0.04
path:hsa05412	Arrhythmogenic right ventricular cardiomyopathy (ARVC)	0	2	7	2	18	0.29	0.71	0.03
path:hsa05414	Dilated cardiomyopathy	0	9	31		4	36	0.04	0.96
path:hsa05416	Viral myocarditis	1	8	28		4	36	0.03	0.97

* 0 indicates biological pathway and 1 indicates disease pathway.

Table S5. Probability that each of the 40 chemicals is a member of each of the 7 clusters in Figure 4.

**Chemical Name**	**Cluster Number**	**Cluster Membership**						
		**Cluster 0**	**Cluster 1**	**Cluster 2**	**Cluster 3**	**Cluster 4**	**Cluster 5**	**Cluster 6**
1,3-butadiene	2	0	0	1	0	0	0	0
3,3'-dichlorobenzidine	6	0.045	0	0	0	0	0.097	0.858
adriamycin	T	0	0	0	0	0	0	1
bcnu	6	0	0	0	0	0.027	0.098	0.875
benzene	6	0	0	0	0	0	0	1
busulfan	6	0	0	0.001	0	0.485	0.017	0.497
ccnu	6	0.012	0.249	0.176	0	0.007	0.16	0.396
chlorambucil	6	0	0	0	0	0	0.025	0.975
chloramphenicol	6	0	0	0	0	0	0.005	0.995
cisplatin	6	0	0	0	0	0	0	1
cyclophosphamide	5	0	0.004	0.005	0	0	0.979	0.012
ddt	6	0	0	0	0	0	0.005	0.995
ethylene_oxide	2	0.003	0	0.948	0	0.023	0.026	0
etoposide	6	0	0	0	0	0	0	1
formaldehyde	6	0.021	0	0	0.003	0.782	0.005	0.189
alpha-hexachlorocyclohexane	6	0.045	0	0	0	0	0.091	0.864
beta-hexachlorocyclohexane	6	0	0	0	0	0.015	0	0.985
delta-hexachlorocyclohexane	6	0.009	0.06	0	0.088	0	0.029	0.814
lindane	6	0	0	0	0.002	0.055	0.142	0.801
me_ccnu	6	0.001	0	0	0	0	0.093	0.906
melphalan	6	0	0	0.004	0	0.19	0.175	0.631
nitrogen_mustard	6	0	0	0	0	0	0.045	0.955
oxymethalone	6	0	0	0	0	0	0.028	0.972
propylthiouracil	6	0.041	0	0.542	0.336	0	0	0.081
styrene	5	0	0	0.143	0	0.09	0.47	0.297
teniposide	5	0	0.016	0.037	0.179	0.021	0.735	0.012

Table S5. *Cont.*

**Chemical Name**	**Cluster Number**	**Cluster Membership**						
		**Cluster 0**	**Cluster 1**	**Cluster 2**	**Cluster 3**	**Cluster 4**	**Cluster 5**	**Cluster 6**
thiotepa	5	0	0	0	0	0	1	0
trichloroethylene	6	0	0	0	0.001	0	0.028	0.971
vinyl_chloride	4	0	0	0	0	1	0	0
2-NAPHTHYLAMINE	6	0	0	0	0.334	0	0.001	0.665
4-AMINOBIPHENYL	3	0	0	0	1	0	0	0
AZATHIOPRINE	6	0	0	0	0	0	0	1
BENZIDINE	6	0	0	0	1	0	0	0
CYCLOSPORINE	6	0.899	0	0	0	0.033	0	0.068
DIETHYLSTILBESTROL	6	0	0	0	0.028	0.012	0.043	0.917
PHENACETIN	1	0	1	0	0	0	0	0
SULFUR_MUSTARD	6	0	0	0	0	0	0.001	0.999
TAMOXIFEN	6	0	0	0	0	0	0	1
TCDD	0	1	0	0	0	0	0	0
TETRACHLOROETHYLENE	6	0	0	0	0.001	0	0.306	0.693

Table S6. Grouping of chemicals into 5 groups for the five-fold cross-validation performed in the One-class SVM analyses.

Chemical group (fold) no.	Chemical Name
1	1,3-butadiene<br>3,3'-dichlorobenzidine<br>adriamycin bcnu<br>benzene busulfan
2	ccnu<br>chlorambucil<br>chloramphenicol cisplatin<br>cyclophosphamide ddt<br>ethylene_oxide
3	etoposide<br>formaldehyde<br>alpha-hexachlorocyclohexane<br>beta-hexachlorocyclohexane<br>delta-hexachlorocyclohexane
4	lindane<br>me_ccnu<br>melphalan<br>nitrogen_mustard<br>oxymethalone<br>propylthiouracil
5	styrene teniposide<br>thiotepa<br>trichloroethylene<br>vinyl_chloride

© 2012 by the authors; licensee MDPI, Basel, Switzerland. This article is an open access article distributed under the terms and conditions of the Creative Commons Attribution license (http://creativecommons.org/licenses/by/3.0/).
